# Mental health supported accommodation services: a systematic review of mental health and psychosocial outcomes

**DOI:** 10.1186/s12888-018-1725-8

**Published:** 2018-05-15

**Authors:** Peter McPherson, Joanna Krotofil, Helen Killaspy

**Affiliations:** Division of Psychiatry, Faculty of Brain Sciences, UCL, 6th Floor, Maple House, 149 Tottenham Court Road, London, W1T 7NF UK

**Keywords:** Supported accommodation, Supported housing, Rehabilitation, Recovery, Effectiveness

## Abstract

**Background:**

Post-deinstitutionalisation, mental health supported accommodation services have been implemented widely. The available research evidence is heterogeneous in nature and resistant to synthesis attempts, leaving researchers and policy makers with no clear summary what works and for whom. In this context, we undertook a comprehensive systematic review of quantitative studies in order to synthesise the current evidence on mental health and psychosocial outcomes for individuals residing in mental health supported accommodation services.

**Methods:**

Using a combination of electronic database searches, hand searches, forward-backward snowballing and article recommendations from an expert panel, 115 papers were identified for review. Data extraction and quality assessments were conducted, and 33 articles were excluded due to low quality, leaving 82 papers in the final review. Variation in terminology and service characteristics made the comparison of service models unfeasible. As such, findings were presented according to the following sub-groups: ‘Homeless’, ‘Deinstitutionalisation’ and ‘General Severe Mental Illness (SMI)’.

**Results:**

Results were mixed, reflecting the heterogeneity of the supported accommodation literature, in terms of research quality, experimental design, population, service types and outcomes assessed. There is some evidence that supported accommodation is effective across a range of psychosocial outcomes. The most robust evidence supports the effectiveness of the permanent supported accommodation model for homeless SMI in generating improvements in housing retention and stability, and appropriate use of clinical services over time, and for other forms of supported accommodation for deinstitutionalised populations in reducing hospitalisation rates and improving appropriate service use. The evidence base for general SMI populations is less developed, and requires further research.

**Conclusions:**

A lack of high-quality experimental studies, definitional inconsistency and poor reporting continue to stymie our ability to identify effective supported accommodation models and practices. The authors recommend improved reporting standards and the prioritisation of experimental studies that compare outcomes across different service models.

**Electronic supplementary material:**

The online version of this article (10.1186/s12888-018-1725-8) contains supplementary material, which is available to authorized users.

## Background

In Western Europe and North America, the process of de-institutionalisation, defined as “…*the practice of caring for individuals in the community rather than in an institutional environment*” (p.47), has led to a significant increase in community based care for people with severe mental illness (SMI) [1]. Housing-based support, or supported accommodation, operates as a component of the broader mental health ‘care pathway’ and attempts to meet the needs of service users by providing focussed, flexible support. In this context, support aims to address functional impairment, develop practical living skills, improve social functioning and promote recovery and independence [2]. Mental health supported accommodation services have been implemented widely; recent estimates indicate that, in the UK alone, over 60,000 individuals are currently receiving support in these settings [3]. Due to high rates of service use, and expenditure related to staffing, support and infrastructure, this form of intervention is also extremely costly. However, despite the broad implementation of these services and the associated financial burden, little is known about their effectiveness.

Definitional issues are well documented in the literature, and present a significant obstacle to the assessment of the effectiveness of supported accommodation. Both within and between countries, supported accommodation services vary widely in terms of physical structure, staffing arrangements, levels of support, recovery focus, and discharge and move-on policies, contributing to confusion as to what exactly a supported accommodation service ‘looks’ like. Despite these issues being discussed in the literature for over 20 years, there have been few meaningful attempts to address them. As a result, the available literature is heterogeneous in nature and resistant to synthesis attempts, leaving researchers and policy makers with no clear summary of the bigger picture; that is, what works and for whom.

For these reasons, previous attempts to summarise the evidence base have been largely unsatisfactory. O’Malley and Croucher [4] conducted a scoping study of supported accommodation services for people with mental health problems in the UK, aiming to explore evidence for models of good practice. After reviewing 131 studies from an original pool of 2506, the authors concluded that most services are based on the assumption that service users will progress from higher to lower levels of supported accommodation over time, however they could not identify any “*concrete evidence to support any particular model of housing support*” (p.841). Due to the methodology used, the authors did not undertake an assessment of the quality of the publications, thus significantly limiting the validity of the findings. In addition, the study focussed solely on UK papers and is also now more than 10 years old. More recently, a Cochrane Review (initially conducted in 2002, and updated in 2006) [5, 6] compared the efficacy of supported housing schemes, outreach support and standard care. The systematic review considered only randomised controlled trials (RCT) and quasi-randomised trials. A thorough search identified 139 potential studies for inclusion, but after review, none fulfilled the inclusion criteria. While the superiority of RCTs as a ‘gold standard’ for providing evidence for effectiveness is widely acknowledged, there is also a growing argument for considering other quantitative evidence beyond RCT studies. This is particularly salient in cases where RCTs are not possible due to ethical or pragmatic concerns, as is typically the case in in supported accommodation research. The Cochrane review provides a stark comment on the state of the literature in the field, however it does little to describe the existing evidence base.

In light of these observations, it is clear that there is an urgent need to summarise the current evidence as it relates to mental health supported accommodation services. We therefore undertook a comprehensive systematic review of data from quantitative studies in the field, incorporating evidence beyond that derived from RCTs alone. Our aim was to synthesise the current evidence on mental health and psychosocial outcomes for individuals residing in mental health supported accommodation, making comparisons between different models, where the quality of evidence allowed. Our objective was to report findings likely to be of interest to those providing and commissioning mental health supported accommodation, as well as policy makers, and to highlight areas for future research.

This review follows the PRISMA guidelines [7]. The PRISMA checklist and review protocol are available and can be requested directly from the authors.

## Methods

### Inclusion criteria

The review included quantitative studies, and quantitative components of mixed-method studies, that evaluated the effectiveness of supported accommodation on the mental health and psychosocial outcomes of people with mental health problems, published after 1990. No country-based limitations were imposed. The review considered all relevant papers published in Latin alphabet text. Non-English papers were translated prior to data extraction. A separate review was carried out by our research team that focussed on studies that used qualitative methods.

#### Definition of supported accommodation

There is large variation in the terminology used in the supported accommodation sector internationally. For the purpose of this review, we defined mental health supported accommodation as any service that provided support, delivered predominately by non-professionally qualified staff, to people with mental health problems living in community-based accommodation, either alone or in shared settings. The components of this definition are common in the literature, and aim to distinguish supported accommodation from specialist, inpatient rehabilitation services, such as community-based rehabilitation units (e.g. ‘ward in the community’) and statutory mental health teams, where staff are required to possess appropriate professional qualifications. All studies that investigated mental health and psychosocial outcomes in these settings were included. Cost-effectiveness papers, and studies examining specific interventions within these settings (e.g. token economies), were excluded.

#### Study design

The review examined studies with a broad range of designs, including experimental, quasi-experimental, cohort, case control and observational studies with and without comparison groups. Systematic reviews, clinical guidance, book chapters, conference proceedings and general commentaries or discussion papers were excluded.

#### Population

We included studies that reported outcomes on individuals with a primary mental health diagnosis, aged 18 to 65. Studies reporting outcomes for service users with a primary diagnosis of dementia, learning disability, personality disorder, substance misuse, eating disorder or physical disability were excluded. Studies with an explicit focus on mental health-substance misuse dual diagnosis populations, or those that included a sample with fewer than 50% of participants with a mental health problem were also excluded.

#### Outcomes

Due to the heterogeneity of studies, it was necessary to consider a wide range of mental health and psychosocial outcomes. These were grouped into four categories:

- Service use: Housing stability, including maintaining tenancy/being evicted from tenancy; hospitalisation; imprisonment; psychiatric service contact; move-on to more independent accommodation.
- Mental health and wellbeing: Symptoms of mental illness; death/suicide; self-esteem; mental well-being
- Function: Social functioning (including employment); autonomy; quality of life (QoL); recovery
- Service user satisfaction with care

#### Search strategy

An electronic database search was conducted in January 2015 using MEDLINE, EMBASE, PsycINFO, CINAHL Plus, International Bibliography of the Social Sciences (IBSS), Applied Social Sciences Index and Abstracts (ASSIA), Sociological Abstracts, Web of Science and The Cochrane Library. Terms and concepts relating to ‘mental illness’, ‘supported accommodation’ and key outcomes, such as quality of life, housing retention and social functioning, were combined with MeSH terms, subject headings or thesaurus terms (depending on database). Searches were conducted again in June 2017 to ensure comprehensiveness. Limits relating to age (18–65 years) and publication date (> 1990) were applied. The original search strategy, organised according to database, is provided in Additional file 1.

Four journals returning the highest number of retained articles in the electronic search were selected for hand-searching: Community Mental Health Journal, Psychiatric Services, Psychiatric Rehabilitation Journal and International Journal of Social Psychiatry. Two authors (PM, JK) reviewed all issues of these journals to identify any articles not retrieved through the formal search. An expert panel, comprised of the Programme Management Group of the Quality and Effectiveness of Supported Tenancies (QuEST) research project (a national programme of research into mental health supported accommodation funded by the National Institute of Health Research [NIHR], Ref. RP-PG-0610-10,097), were also asked to provide key publications. Reference lists from six key papers [8–13] were also reviewed in order to identify any articles missed through the other search strategies.

After the initial database search results were collated, and duplicates omitted, a relevance review of 10% of articles (*n* = 1066) was conducted by the reviewers (PM, JK) to ensure fidelity to the inclusion criteria. There was 2.5% discrepancy between the two raters (*n* = 27 articles). These 27 publications were reviewed and discussed until consensus regarding inclusion was reached.

#### Data extraction

A data extraction form was created to facilitate recording of key study information. Data extraction was carried out by two researchers (PM, JK), with the following information recorded from each included article:

- Article characteristics: Country of origin; language; population; subject and context.
- Study characteristics: Aims of the study; study design; theoretical framework; outcomes; methods; participant numbers by group (N); participant eligibility; sampling method; recruitment procedures; data collection procedures; data analysis.
- Findings: Reported results; interpretation of results; summary of findings; recommendations; policy and practice implications.

For non-English language articles, researchers at UCL that were native-speakers were employed to extract the data. JK and PM instructed these individuals on the process of data extraction.

#### Quality assessment

Quality appraisal of articles was carried out to assess bias and inform the relative weighting of results. The Quality Assessment Tool for Quantitative Studies (QATQS) [14] was used to assess article quality; the tool is recommended for use in systematic reviews by Deeks and colleagues [15] and displays acceptable psychometric properties [14]. The QATQS assesses the methodological strength of a study across eight domains (selection bias; study design; confounders; blinding; data collection methods; withdrawals and dropouts; intervention integrity; and analysis) and provides a global quality rating of ‘strong’ (high quality), ‘moderate’ (moderate quality) and ‘weak’ (low quality). Due to the scoring system, it is possible for well-designed and executed non-experimental studies to be rated as high quality (e.g. cohort analytic, case control, and interrupted time series rated as ‘moderate’ in the design domain). Any papers assessed as ‘weak’ were excluded from the synthesis (see below).

#### Data synthesis

Due to the heterogeneity of the literature, a narrative synthesis method was employed. The synthesis was structured using guidelines published by Popay and colleagues [16], and included developing a preliminary synthesis, exploring the relationships in the data, and assessing the robustness of the final synthesis product.

## Results

### Descriptives

**Fig. 1:**
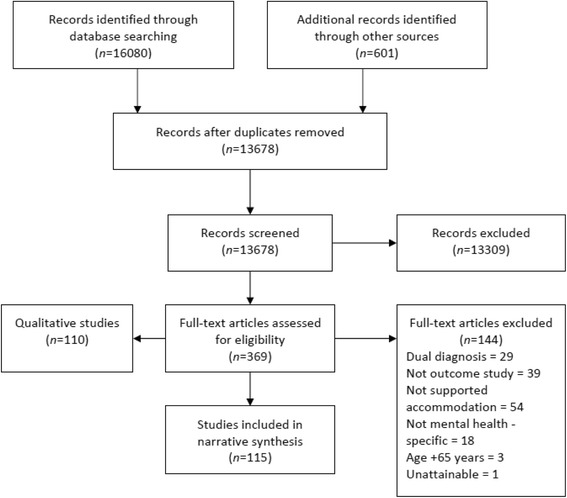
PRISMA diagram: Retrieval process

The initial return comprised of 16,080 articles from electronic databases, and 601 articles from hand-searches and snowballing. After the removal of duplicates, and the application of inclusion-exclusion criteria, the final sample consisted of 115 articles. A PRISMA diagram, illustrating the retrieval procedure and returns, is presented in Fig. 1. The majority of the retrieved papers focussed on American populations, with smaller numbers considering Canadian, United Kingdom, Italian, Australian and German contexts (see Table 1). In terms of experimental design, the review considered cohort (*n* = 85), quasi-experimental (*n* = 15), randomised control (*n* = 11) and case-control (*n* = 4) studies. Quality assessment, using the QATQS, indicated that the majority of studies were rated as moderate quality (*n* = 62), with a smaller number rated as high quality (*n* = 20). In total, 33 studies were assessed as low quality and, in line with our protocol, excluded from the synthesis.

**Table 1:** Retrieved papers by country of origin

Country	Number of papers
USA	50
Canada	14
UK	11
Italy	8
Australia	6
Germany	6
Denmark	3
Hong Kong	3
Israel	3
Japan	3
Holland	2
Albania	1
Finland	1
Greece	1
India	1
New Zealand	1
Taiwan	1
Total	115

Initially, the review was intended to assess and contrast outcomes according to supported accommodation service types. However, the significant variation in terminology and service characteristics in the reviewed papers made this approach unfeasible. Consequently, we examined the characteristics of individual studies, in line with guidance by Popay and colleagues [16], and identified specific study populations. Two clear sub-groups emerged; homeless individuals with mental illness, and former patients of large hospitals that had been resettled in the community. The remaining papers considered supported accommodation for non-specified mentally ill populations. Based on these characteristics, it was decided to present the findings according to the following population sub-groups: ‘Homeless’, ‘Deinstitutionalisation’ and ‘General Severe Mental Illness (SMI)’. These three groups of studies are described in the sub-sections below. See Additional file 2 for a summary table of all reviewed studies.

### Homeless

This group of papers comprised of studies examining outcomes of supported accommodation focussed on homeless individuals with SMI. These studies were typically from the USA or Canada, and included permanent supported housing projects (such as Housing First [HF]) and the ‘HUD-VASH’ program (Department of Housing and Urban Development - Veterans Affairs Supportive Housing Program), which caters to military veterans in the USA. These studies are unique, not only for their population-focus but also for the type of supported accommodation examined; the bulk of the ‘homeless’ papers examined the permanent supported housing approach which aims to support homeless individuals to achieve housing stability or permanent accommodation, as well providing support with mental health issues. Specifically, this approach “*provides individuals with immediate housing, client choice is emphasized in every aspect of treatment, housing is separated from treatment, and a harm reduction approach is followed*” [13] (p.2). It contrasts with traditional continuum models whereby service users progress through ‘levels’ of support (of lessening intensity), with the intention of achieving stable, permanent housing as an end-point.

#### Study design, quality and outcomes

The ‘homeless’ group comprised of the largest number of examined studies; 40 papers in total were reviewed. Of this, 11 were rated as high quality [8, 13, 17–24, 44] and 20 moderate quality [9, 25–43]. Nine papers were omitted from the synthesis due to low quality. Of the retained papers, the majority were cohort studies (*n* = 16), followed by RCTs (*n* = 8), quasi-experimental studies (*n* = 6), and a single case control study (*n* = 1) (see Additional file 2 for detail). The outcomes assessed varied, but reflect the high number of HF studies and their primary foci; the most common outcomes were housing stability, service use, symptoms of mental illness, substance misuse, social functioning, and QoL.

#### Supported accommodation types

The structure of the examined supported accommodation services varied considerably, as did the detail in which they were reported. The majority of the papers on homeless supported accommodation services were based on the permanent supported housing model [9, 13, 17, 18, 21–23, 25–27, 30, 31, 33, 36–44]. Projects adhering to this model included, among others, HF [13, 17, 18, 26, 27, 31–33, 36, 37, 40, 41], Pathways to Housing [34, 44], At Home/Chez Soi [42], and projects using Section 8 housing certificates [39]. The principles of permanent supported housing are implemented in various forms; accommodation types include group homes [24, 35, 43], individual apartments [24, 35, 38, 43], community residences (residences in buildings with single or shared rooms, or studio apartments, and common dining, meeting, and services space) [21]. The permanent supported housing projects also varied according to intensity and nature of support (including intensive case management, assertive community treatment and different levels of on-site staff support), level of integration between housing and mental health service providers, fidelity to the principle of separation between housing and treatment, and restrictions around sobriety [9]. Traditional housing available to homeless populations with SMI, such as homeless shelters [29], nursing homes [20], board and care homes [28] and residential care facilities [35] were used as comparison conditions in some studies.

##### Housing stability

In total, 19 studies of supported accommodation for homeless populations assessed housing stability as an outcome, comprising 7 high quality [8, 18, 19, 21–23, 44] and 12 moderate quality studies [9, 25, 26, 30–35, 37, 39, 42]. Supported accommodation appears to be effective in promoting housing retention for homeless adults with mental illness; high quality studies consistently reported high rates of housing retention, with 37 to 84% of participants still housed at follow-up (6 months to 5 years) [19, 21, 23, 44, 45]. Moderate quality studies generally supported these findings, reporting high rates of housing stability [9, 34, 35], reductions in nights spent homeless [26, 33, 37], and increases in nights spent in own apartment [26, 42].

A number of factors appear to be related to housing stability including gender, with women more likely to be housed and less likely to be evicted [18, 30], age (being older associated with longer tenure) [19, 34] and income [9], with higher income increasing the probability of a ‘positive’ move-on. Other related factors include access to housing subsidies [39], neighbourhood quality [9], supportive relationships with support staff [9], case manager support to access appropriate benefits [18], self-harm behaviours [30], chronic pain/illness [30], improvements in housing problems [8] and past [9, 19] and current substance misuse [30]. Intensity of case management [39] and service users’ preference for housing type and satisfaction with support were not found to be predictive of housing stability [31, 32].

##### Service use

Eight studies of supported accommodation for homeless populations examined the association between the supported accommodation service and appropriate service use (two high quality studies [8, 21] and six moderate quality studies [28, 29, 35–37, 42]). There was generally consistent evidence that supported accommodation for homeless individuals lead to an increased use of appropriate support services, such as outpatient clinics and medication visits [36, 37], a reduction in hospitalisations [28], homeless shelter use [29] and the use of crisis services [21] for adult homeless populations. In contrast, a single moderate quality study [42] found no difference in hospital days or emergency service use between HF participants and those receiving treatment as usual (TAU). One high quality study found a positive association between neighbourhood quality and length of hospital admissions, and between housing problems and service needs [8]. One moderate quality study [35] demonstrated a significant relationship between housing stability and appropriate service use.

##### Symptoms of mental illness

Eight studies of homeless supported accommodation services investigated mental health symptoms (five high quality [13, 21, 23, 44, 45] and three moderate quality studies [32, 33, 42]). Symptom assessment methods varied (across all studies in this review), and included clinical assessments, staff-rated and self-report instruments. All high quality studies suggested that supported accommodation was associated with significant improvements in, or stability of, mental health symptoms [13, 21, 23, 44, 45]. No deterioration in symptoms was reported. Results of moderate quality studies were inconsistent; one study found no differences in symptoms over time between HF and TAU groups [42], while another reported that improvements in symptoms were not consistently observed and, where they did occur they were slight [33].

One high quality study [13] reported all findings, including symptoms, in terms of “expected trajectory”, referring to an improvement or no change in key variables. This presented a challenge in interpreting the results, as improvements, and lack of change in outcomes, were not presented separately. Although this study suggested that 71% of participants followed the expected trajectory for improvements in symptomology, no data were provided to distinguish what proportion of this group improved, or did not change. This observation pertains to all subsequent reporting of findings from this study.

One study reported a greater improvement in psychiatric symptoms in supported housing settings where mental health services are integrated (see descriptions above) [23]. Paradoxically, one high quality study found that a diagnosis of alcohol or substance abuse or dependence was associated with a reduction in psychiatric symptoms [13].

##### Substance use

In total, six homeless supported accommodation service studies examined the association between the supported accommodation and substance use (four high quality [13, 23, 44, 45] and two moderate quality studies [33, 42]). There was no clear evidence that supported accommodation was associated with reduction in drug and alcohol use for homeless populations. The majority of high quality studies reported no change in this outcome over time [23, 44, 45]. A single high quality study reported improvement or no change in this outcome, with 72% of the sample meeting the expected trajectory for change [13]. A single moderate quality paper reported a small but significant reduction in ratings of alcohol abuse, and in the proportion of participants who reported illicit substance misuse [33]. Similarly, a moderate study reported a significant reduction number of days experiencing alcohol-related problems at 12 and 24 months, and in the amount of money spent on alcohol at 24 months, in the HF group compared to TAU [42]. Although we did not find evidence that supported accommodation was associated with reduction in substance misuse, it is important to note that it was not associated with any increase amongst homeless individuals either.

##### Social functioning, family support and community integration

Across the seven studies (two high quality [13, 45], five moderate quality [31, 33, 38, 41, 42]) that examined this outcome, there was inconsistent evidence to suggest that supported accommodation was associated with improvements in social functioning, family support or community integration. A single high quality study reported significant improvement in satisfaction with family relationships, perceived availability of family and frequency of family interactions over time [45]. Study 13 provides perhaps the strongest evidence in support of improvements in these outcomes: 60, 62 and 67% of participants followed the ‘expected trajectory’ of improvement for physical community integration (actual participation in community activity and use of community resources), psychological community integration (how an individual perceives themselves as a member of their community) and community functioning respectively [13]. However, as described previously, the method of reporting in this study limits our ability to confidently interpret the findings. One study [41] demonstrated significant improvements in psychological community integration at 12-month follow-up for HF participants, when compared to TAU, however they found no change between groups or over time in physical community integration. Similar results were reported in another moderate quality paper [42]. A moderate quality study found a small but statistically significant increase in overall community participation over time [33], however, for nine of the 18 activities assessed, there was no significant change; the authors reported that “*Participants …remained socially isolated and showed limited improvement in other domains of social integration”* (p.427).

##### Quality of life

The data were inconsistent in demonstrating any association between supported accommodation for homeless individuals and QoL. In total, nine papers reported on this outcome (four high quality studies [13, 21, 23, 44] and five moderate quality studies [27, 31, 37, 40, 42]). Amongst the high quality papers, one study reported improvement in QoL at 18-months [23], one reported 66% of participants meeting the “expected trajectory” for change in QoL [13], and one reported no change over time [21]. Another high quality study reported no significant difference in QoL between participants receiving HF and controls at six months follow-up, however, baseline data were not reported so change over time could not be assessed [44]. One moderate quality study demonstrated initial improvement in QoL in a HF group, compared to TAU, however the effect was not sustained, with no group differences in QoL observed at 24 month follow-up [42]. Data from other moderate quality studies suggested that supported accommodation is associated with improvements in QoL for homeless adults [27, 40], but the experimental design of another moderate quality study did not allow examination of change over time [37]. Time in independent housing was significantly associated with QoL [27]. The same study showed QoL was negatively associated with severity of symptoms but it was not associated with participation in community activities [27]. Another study found service users having choice over living environment predicted QoL at six and 12-month follow up, albeit weakly [31].

### Deinstitutionalisation

The ‘deinstitutionalisation’ subgroup of papers is comprised of studies examining outcomes of people previously residing longer term in large mental hospitals who were resettled in the community following the closure of these institutions and the development of community based mental health services. These individuals were typically older, with a long illness history and had been in hospital for an extended period of time.

#### Study design, quality and outcomes

In total, 35 deinstitutionalisation papers were reviewed; five were rated as high quality [46–50] and 23 as moderate quality [51–73]. Seven were omitted from the synthesis due to a low quality rating. The included articles were typically cohort studies (*n* = 24), with a smaller number of quasi-experimental (*n* = 2) and a single case-control (*n* = 1) and randomised controlled trial (*n* = 1) (see Additional file 2 for detail). Common outcomes assessed for this group were rates of hospitalisation, symptoms, social functioning, employment and QoL.

#### Supported accommodation types

Due to the naturalistic nature of the deinstitutionalisation process and the associated research, the majority of the studies were uncontrolled, and often considered a range of community-based accommodation settings within a single study; this contributed, at times, to descriptions of services that lacked detail. Identifiable service types included nursing homes [47, 56, 65], residential care units or high-support hostels with 24 h staffing [46–48, 51, 52, 54, 55, 57, 59, 60, 62, 63, 66], supported group homes [48, 49, 55, 56, 61, 64, 67], apartments with flexible support [46, 48, 56, 60], halfway houses [50, 53, 58, 68, 71, 73], and boarding/rooming houses [56, 59, 61]. Some studies refer to ‘supported housing’ or ‘supported accommodation’ services without providing further detail [70, 72].

##### Rates of hospitalisation

Twelve studies, including one high quality [48] and 12 moderate quality studies [51–53, 56, 58, 60, 63, 67–69, 71, 72], examined rates of hospitalisation as an outcome. Unfortunately, the single high quality study did not report data assessing change over time [48]. Amongst the moderate quality studies, however, supported accommodation appeared to be related to reduce rates of hospitalisation [51, 53, 60] and duration of hospital admissions [53, 69, 71] over time. There was, across almost all studies, clear evidence of high rates of rehospitalisation; a number of moderate quality studies [52, 53, 56, 58, 63, 67, 68, 72] indicated between 35 and 87% of participants required inpatient treatment at least once during the follow-up period (range 4–10 years). The single high quality study reported a rehospitalisation rate of 22% during the follow-up period [48]. There is some evidence to suggest that participants from more highly supported settings were more likely to hospitalised, than those in more independent settings [56].

##### Symptoms

There was strong evidence for improvement or a lack of deterioration in the severity of symptoms of mental illness for patients who were discharged to community-based supported accommodation settings from large institutions. In total, 15 studies examined this outcome, including four high quality [46–49] and 11 moderate quality studies [52, 54, 56, 57, 62, 63, 65, 66, 70, 72, 73]. The majority of high quality studies reported improvements in symptoms over time [46, 47, 49]. Improvement in positive symptoms was most common [47, 50]. Findings from the moderate quality papers were less consistent; most reported no change [52, 62, 63, 65, 66, 70, 72, 73], or mixed results [54, 56, 57].

A number of high and moderate quality studies seemed to indicate that more restrictive settings, such as nursing homes were associated with poorer outcomes, with some demonstrating a worsening of symptoms in patients discharged to these environments [47, 73].

##### Social functioning

Fifteen studies examined social functioning as an outcome, including four high quality [47–50] and 11 moderate quality studies [52, 54–58, 62, 63, 65, 66, 70]. Evidence for an association between supported accommodation and improvement in social functioning amongst the ‘deinstitutionalisation’ sub-group was mixed, although findings suggested a trend toward improvement. Two high quality studies reported significant improvements in performance of socially expected activities [50], social competence and social interest [49], and a significant reduction in behavioural problems, such as hostility, over/under activity and inappropriate sexual behaviour [50]. Two high quality studies, however, found no improvements in social functioning over time [47, 48]. Data from the moderate quality studies contributed to the mixed picture; while some studies reported significant improvements over time [54, 63, 65], the evidence was complicated by poor methodologies and, occasionally, a lack of inferential data [55, 62]. In addition, several moderate quality studies reported no change in this domain [52, 65, 66]. Several studies showed no change in the size of social networks over time [57, 70], while others demonstrated a reduction in the frequency of contacts with family and friends, with a parallel increase in contacts with fellow residents [58]. Similar to the findings reported previously for symptoms, one study reported a deterioration in global social adjustment in individuals discharged to more restrictive settings, such as psychiatric nursing homes [47].

##### Employment

There was some evidence supporting an association between supported accommodation and employment in the deinstitutionalised population, however the number and quality of studies was limited. Employment rates ranged from 0 to 17% in the three moderate quality studies that assessed this outcome [56, 68, 72]. One moderate study reported an unspecified reduction in rates of unemployment at the time of discharge [53]. No high quality papers examined this outcome.

##### Quality of life

A relatively small proportion of the reviewed studies examined QoL as an outcome in the deinstitutionalised subgroup; in total, eight studies, including two high quality [47, 50] and six moderate quality studies [54, 61, 63, 66, 72, 73], examined this variable. There was a lack of clear evidence for any improvement in QoL over time for this population but most studies reported that it remained stable. The high quality papers suggested an inverse relationship between QoL and restrictiveness of setting, with studies reporting significant reduction in QoL for patients discharged to psychiatric nursing homes [47, 50]. Similarly, one moderate quality paper found that moving into ‘inappropriate’ residential settings resulted in a deterioration in QoL [61]. The lack of association between supported accommodation and subjective QoL over time reported in the high quality papers was consistent with the evidence from the moderate quality papers [54, 72, 73].

### General SMI

Although this group is largely defined by non-inclusion in the Homeless and Deinstitutionalisation groups, a number of characteristics make it distinct. The General SMI group represents service users of the post-deinstitutionalisation rehabilitation pathway; generally, they present with complex needs associated with psychotic illness and will have entered the supported accommodation system through various pathways (e.g. referred from acute inpatient units, deteriorated after independent community living, or moved-on from community-based rehabilitation units / forensic services etc). Importantly, as opposed to the deinstitutionalisation group, they will generally not present with the clinical, social and behavioural impairments associated with long-term hospitalisation.

#### Study design, quality and outcomes

In total, 40 general SMI papers were reviewed. The majority were of moderate quality (*n* = 19) [74–91, 96], with a smaller number rated as high quality (*n* = 4) [92–95]. However, 17 papers were not included in this synthesis due to low quality. As with the previous subgroups, the majority of the studies were cohort designs (*n* = 18), with one quasi-experimental (*n* = 1) and one matched case control study (*n* = 1) (see Additional file 2 for detail). There was a wide variety of outcomes assessed in these papers, however the most common were rates of hospitalisation, symptoms, social functioning, and QoL.

#### Supported accommodation types

For this group of papers, service types ranged from intensive congregate residential care settings, with 24 h staffing [76, 78–80, 87, 89, 93, 95], to less intensively supported accommodation, including group homes and supervised individual apartments (e.g. staffed 9 am-5 pm daily) [74, 78, 81, 82, 90, 92–94], to individual tenancies with outreach support (staff based off-site) [75, 81, 83, 87, 88, 90, 96]. Other service descriptions lacked detail and were unable to be confidently categorised, including “sheltered housing” [77], “sheltered-care facilities” [86], “community-based housing” [91], “transitional, high-expectation, sheltered care environment” [85] and “support house” [84].

##### Rates of hospitalisation

Twelve papers, including two high quality [92, 94] and ten moderate quality studies [74, 77, 78, 81, 82, 84, 88–90, 96], reported data relating to changes in rates of hospitalisation. The findings were mixed. Generally, it appeared that supported accommodation was associated with a reduction in days spent in hospital [78, 81, 82, 89, 90], and duration of hospital stays [96], over time. One study demonstrated a significant reduction in mean number of hospital and crisis centre admissions from pre-entry into a residential program to one-year post-discharge [89]. Similarly, another moderate quality study demonstrated a significant reduction in mean number of days in hospital from baseline year (pre-entry) to the first year in community living arrangement facilities [78]. In spite of these positive findings, other studies, including a single high quality article [94], reported no change in this outcome over time [74, 88]. One high quality study found that specialist case management, which was reserved for SUs with a history of repeated hospitalisations, was the only predictor of future hospitalisations [92], while another moderate quality study found that severity of symptoms predicted both hospitalisations and duration of stay [77].

##### Symptoms

Nine papers assessed changes in symptoms over time (two high quality [94, 95] and seven moderate quality studies [74, 76, 80, 82, 84, 86, 88]. There was mixed evidence for an association between supported accommodation and symptoms in general SMI populations. Although some moderate quality papers demonstrated significant improvements in symptoms over time [82, 84, 95], a number, including a single high quality study, reported no change [86, 94], or a worsening of symptoms [74, 80] amongst this group. In examining change over time, one moderate quality study showed a significant improvement in symptoms for a small sub-group of participants who had been discharged from a residential facility, but no change for those participants who had remained in place [76]. One study demonstrated improvement in depression and anxiety over time, however this was assessed using a scale assessing social behaviours as opposed to a symptom inventory [88].

##### Social functioning

In total, 12 general SMI studies examined the association between supported accommodation and social functioning, including one high quality [94] and eleven moderate quality papers [74–76, 79, 82, 83, 85–88, 96]. Again, the findings related to this outcome are best described as mixed. Some moderate quality studies demonstrated significant improvements in social integration [82] and community participation [96], increases in friendships and supportive relationships [82, 83] and reductions in social disability over time [79]. However, the majority of studies found no change in key social variables over time, such as social functioning [74, 76, 82], social networks [74, 75, 82] and satisfaction with social support [94]. One study, examining the effectiveness of outreach support, found a significant reduction in the size of social networks over time [88]. Another study reported a reduction in independent social functioning and an increase in assisted social functioning over time [86]. In contrast, a single moderate quality study found that SUs receiving home care reported significantly higher levels of social activity compared to SUs residing in half-way house [87]. Severity of symptoms and number of hospitalisations was found, in one study, to negatively influence the quality of social networks [85].

##### Quality of life

Six moderate quality papers [74, 79, 80, 82, 87, 88] examined changes in QoL with the general SMI group. The findings were mixed; half reported a significant improvement in QoL over time [79, 82] or a non-significant trend towards improvement [88], and half indicated no change [74, 80, 87], suggesting a limited, or inconsistent, relationship between supported accommodation and this outcome. In one study, QoL was positively associated with specific life skills, including budgeting, self-care, ability to go out, and work performance [82].

## Discussion

This systematic review attempted to synthesise the literature on mental health and psychosocial outcomes associated with mental health supported accommodation services. Despite the initial aim of comparing and contrasting outcomes across supported accommodation models, the wide variation in accommodation services, in terms of structure, staffing and related variables, required us to group our findings by population sub-groups.

### Homeless

The strongest evidence for supported accommodation comes from research with homeless mentally ill populations and the permanent supported housing model. Studies in this area demonstrate consistent evidence for improvements in housing retention and stability, and appropriate use of clinical services over time. There is also some indication that this form of support for this group is associated with improvements in symptoms, QoL and social functioning, but this evidence is inconsistent. The majority of studies reviewed found no change in substance use over time. As stated by Tsermberis [97], “*Housing First and other supportive housing interventions may end homelessness but do not cure psychiatric disability, addiction, or poverty*” (p.52). These findings are in line with the conclusions of a recent review of HF [98].

Although the permanent supported housing approach has been shown to be effective in some domains, the intervention specifically targets mentally ill homeless populations, and the characteristics of the studied cohorts make generalising to other mental health populations troublesome. First, many of the samples used in these studies have relatively low rates of serious psychiatric illness. In the current review, we utilised a > 50% with psychiatric diagnosis cut-off point to ensure we included appropriate studies. However, even with this approach, it remains difficult to confidently apply the synthesised findings to other groups of people with mental health problems. Second, the presence of long term homelessness amongst the target population conflates the findings when considering the applicability to general SMI populations. It remains difficult to establish whether positive changes in psychosocial outcomes are attributable to intervention components that impact on homelessness (such as housing), or mental health (such as medication management) or both. Third, participants in the permanent supported housing studies typically present with higher rates of drug and alcohol use than comparable, non-homeless samples. There is danger that, due to the large and growing evidence base for these services, policy makers will attempt a wholesale import of the permanent supported housing model for use with psychiatric populations, without a reliable evidence base.

### Deinstitutionalisation

Research on outcomes in supported accommodation for deinstitutionalised populations provided good evidence for improvement or non-deterioration in psychiatric symptoms, social functioning and rates of rehospitalisation. There was limited evidence for improvement in QoL and employment. Notably, a number of studies highlighted a consistent association between more restrictive settings and poorer outcomes, across psychiatric, social and QoL outcomes, for this group. Although, these findings are somewhat inconsistent, the threshold of ‘success’ for this population is radically different than for other groups. Due to the severity of clinical presentations and duration of institutionalised care, most researchers and clinicians consider the absence of deterioration as indicative of successful transition to community care. Indeed, one of the greatest challenges of the deinstitutionalisation process was to address the chronic psychiatric, social and behavioural difficulties of patients, while simultaneously maintaining their tenure in the community [1]. Supported accommodation services appear to have contributed to the achievement of these goals; the reported findings, while not consistently demonstrating improvements across domains do, for the most part, highlight stability.

In reality, the deinstitutionalisation ‘story’ has already been told; in most European and north American countries, the deinstitutionalisation process commenced the late 1980s and early 1990s and, as such, the majority of the studies cited in this review are old, or report on longer-term follow-ups. It is generally accepted that community based settings are more humane and offer a better QoL than long term hospitalisation [99]. The transition of people from long-stay wards to community-based care has been successful, and the evidence suggests that this group, for the most part, can be maintained in community settings without any significant deterioration [100]. This is an important and well established finding but does little to guide us in the development and implementation of contemporary supported accommodation services.

### General SMI

The reviewed papers in this group presented less clear evidence between supported accommodation and psychosocial outcomes for general SMI populations. While there was a trend toward reductions in rates of hospitalisation over time, the evidence was mixed with regards to symptoms, social functioning and QoL, with studies variously demonstrating improvement, no-change, or deterioration in these outcomes over time. These findings may reflect the heterogeneous nature of the literature.

This sub-group had the fewest number of studies overall, the fewest number of high-quality papers and the largest number of omitted low-quality studies, yet this population is growing rapidly, reflecting the broad adoption of the supported accommodation model, current approaches to community-based rehabilitation and the rejection of long-term hospitalisation as a form of treatment.. This observation highlights an urgent need for increased research in this area. As mentioned above, there is a genuine danger that due to the growing evidence base, the HF model is applied to this group despite the problems in generalising the research findings.

#### Strengths and limitations

The current review had a number of strengths. We applied a thorough search strategy, utilising a broad date range, and included a range of psychosocial outcomes and a variety of quantitative designs beyond RCTs. These methodological decisions enabled us to be comprehensive in our review and confident in capturing all key outcome studies.

As the reviewed studies relate to supported accommodation only, it must be acknowledged that, by examining the outcomes in relation to subgroups, broader findings related to these groups may have been overlooked. For example, many of the Team for the Assessment of Psychiatric Services (TAPS) studies, and other programmes of research investigating outcomes for deinstitutionalised groups, were not included in this review as they did not explicitly consider supported housing, however data from these studies would have expanded and contextualised the reported findings as they relate to the deinstitutionalisation sub-group. The population-based conclusions, therefore, must be considered strictly in relation to supported accommodation.

As we were unable to examine differences in outcomes across models, the current review cannot comment on their relative merits in relation to outcomes. As described, there is a large variation in housing models; within each of the population sub-groups considered above, service models ranged from independent tenancies with outreach support to high-staffed, congregate residential settings. Inevitably, the characteristics of a service, such as the physical structure, staffing arrangements, levels of support, recovery focus, and discharge and move-on policies, will impact on service user outcomes, possibly beyond the influence of population characteristics. As a result, this review is limited in its ability to fully consider the effectiveness of mental health supported accommodation services.

By comparing services from different national contexts, we aimed to enhance our understanding of the critical components of these interventions and how contextual factors impact outcomes. However, due to the aforementioned variation in service models (evident even within countries), it was difficult to discern the impact of national level factors, such as legislation, funding barriers or statutory responsibilities. The international focus of this review makes it challenging to provide any specific recommendations for local policy makers and commissioners. A more targeted study, focusing on one country or region, would be better suited for this purpose. In line with the recovery approach, however, it is likely that any high-quality supported accommodation provision will comprise of a range of accommodation options, with the delivery of flexible, personalised support.

Finally, as we have considered evidence from non-RCT designs, the data presented herein, even from studies rated as ‘high quality’, should be interpreted with caution.

## Conclusion

The mixed results of this study highlight the heterogeneity of the supported accommodation literature, in terms of research quality, experimental design, population, service types and outcomes assessed. There is some evidence that supported accommodation is effective across a range of psychosocial outcomes, with the most robust evidence showing the effectiveness of the HF model for homeless SMI and for other forms of supported accommodation for deinstitutionalised populations in reducing hospitalisation rates and improving appropriate service use. The evidence base for general SMI populations is less developed, and requires further research. Unfortunately, these broad observations reinforce the conclusions of Chilvers and colleagues [5] in their recent Cochrane review: “*In the absence of evidence of their relative efficacy, decisions on the provision of alternative forms of accommodation and continued support for people with mental illness can only be based on a combination of professional judgement, patient preference and availability*” (p.6).

The intention of the current review was to compare and contrast the effectiveness of various models of supported accommodation, across a range of psychosocial outcomes. However, as noted, this attempt was stymied by the large variation in service models, the lack of definitional consistency and, at times, poor reporting practices in the literature. In order to make assertions regarding the effectiveness of various models of supported accommodation, it is clear that a simple method of service categorisation, based on current reporting practices, is required. A taxonomy that can be applied retrospectively to existing research, and utilised in future studies, would allow effective synthesis of outcome data, facilitate an examination of efficacy and effectiveness, and strengthen follow-up/replication studies [101]. While some attempts have been made to develop a supported accommodation taxonomy [102, 103], these models are complex and have not been widely utilised. Recently, a new, simple classification system for supported accommodation services has been developed (The Simple Taxonomy for Supported Accommodation [STAX- SA] [104]). Future research should consider utilising this tool to synthesise the available effectiveness evidence, comparing service user outcomes across service models.

Mental health supported accommodation services are widely implemented, however, currently we have no clear research base articulating what works and for whom. There is a clear need for high quality effectiveness research, improved reporting standards and consistent and meaningful descriptions of supported accommodation services in the literature. Researchers must prioritise experimental studies that compare outcomes across different service models. These developments should inform and improve mental health commissioning and service development decisions in the future.

## Additional files

Additional file 1: Final Search Strategy. The search strategy used for the systematic review, organised according to database. (DOCX 28 kb)

Additional file 2: Summary table: All included studies. Summary of extracted data from all studies included in the final synthesis. (DOCX 60 kb)

## Abbreviations

HF: Housing First
HUD-VASH: Department of Housing and Urban Development - Veterans Affairs Supportive Housing Program
NIHR: National Institute of Health Research
QATQS: Quality Assessment Tool for Quantitative Studies
QoL: Quality of life
QuEST: Quality and effectiveness of supported tenancies for people with mental health problems
RCT: Randomised controlled trial
SMI: Severe mental illness
TAU: Treatment as usual
