# Supplementary material for: Mental health supported accommodation services: a systematic review of mental health and psychosocial outcomes
Source: BMC Psychiatry. 2018 May 15;18:128. doi: 10.1186/s12888-018-1725-8 (PMC5952646; doi:10.1186/s12888-018-1725-8)
Supplement: Supplementary file 1 — Final Search Strategy. The search strategy used for the systematic review, organised according to database. (DOCX 28 kb) [file 12888_2018_1725_MOESM1_ESM.docx]

Additional File 1

Final Search Strategy

| **MEDLINE (OVID)** | | **EMBASE (OVID)** | | **CINAHL Plus (EBSCO)** | |
| --- | --- | --- | --- | --- | --- |
| *N* | *Terms* | *N* | *Terms* | *N* | *Terms* |
| 1 | exp Mental Disorders/ | 1 | exp Mental Disorders/ | S1 | (MH "Mental Disorders+") |
| 2 | Mental Health/ | 2 | Mental Health/ | S2 | (MH "Mental Health") |
| 3 | ((psychol* or psychiat* or mental*) adj (illness* or disorder* or problem* or disease* or disab*)).tw. | 3 | ((psychol* or psychiat* or mental*) adj (illness* or disorder* or problem* or disease* or disab*)).tw. | S3 | TI (psychol* or psychiatr* or menta*l) W0 (illness* or disorder* or problem* or disease* or disab*) OR AB (psychol* or psychiatr* or mental*) W0 (illness* or disorder* or problem* or disease* or disab*) |
| *4* | *1 or 2 or 3* | *4* | *1 or 2 or 3* | *S4* | *S1 OR S2 OR S3* |
| 5 | residential facilities/ or assisted living facilities/ or group homes/ or halfway houses/ or nursing homes/ | 5 | residential facilities/ or assisted living facilities/ or group homes/ or halfway houses/ or nursing homes/ | S5 | (MH "Residential Facilities") OR (MH "Assisted Living") OR (MH "Halfway Houses") OR (MH "Nursing Homes") |
|  |  |  |  | S6 | TI (“group home*”) OR AB (“group home*”) |
|  |  |  |  |  |  |
| 6 | Residential Treatment/ | 6 | Residential Treatment/ | S7 | TI ("Residential Treatment") OR AB ("Residential Treatment") |
| 7 | (residential adj (care or rehab* or service* or home*)).tw. | 7 | (residential adj (care or rehab* or service* or home*)).tw. | S8 | TI (residential W0 care or rehab* or service* or home*) OR AB (residential W0 care or rehab* or service* or home*) |
| 8 | ((support* or shelter* or assist*) adj (hous* or home* or accom* or living or tenanc*)).tw. | 8 | ((support* or shelter* or assist*) adj (hous* or home* or accom* or living or tenanc*)).tw. | S9 | TI (support* or shelter* or assist*) W0 (hous* or home* or accom* or living or tenanc*) OR AB (support* or shelter* or assist*) W0 (hous* or home* or accom* or living or tenanc*) |
| 9 | ((floating or visiting) adj (support or outreach)).tw. | 9 | ((floating or visiting) adj (support or outreach)).tw. | S10 | TI (floating or visiting) W0 (support or outreach) OR AB (floating or visiting) W0 (support or outreach) |
| 10 | outreach.tw. | 10 | outreach.tw. | S11 | TI (outreach) OR AB (outreach) |
| *11* | *5 or 6 or 7 or 8 or 9 or 10* | *11* | *5 or 6 or 7 or 8 or 9 or 10* | S12 | *S5 OR S6 OR S7 OR S8 OR S9 OR S10 OR S11* |
| 12 | exp Hospitalization/ | 12 | exp Hospitalization/ | S13 | (MH "Hospitalization+") |
| 13 | Death/ | 13 | Death/ | S14 | (MH "Death") |
| 14 | exp Suicide/ | 14 | exp Suicide/ | S15 | (MH "Suicide+") |
| 15 | personal satisfaction/ | 15 | personal satisfaction/ | S16 | (MH "Personal Satisfaction") |
| 16 | exp Patient Satisfaction/ | 16 | exp Patient Satisfaction/ | S17 | (MH "Patient Satisfaction") |
| 17 | "Quality of Life"/ | 17 | "Quality of Life"/ | S18 | (MH "Quality of Life") |
| 18 | Social Adjustment/ | 18 | Social Adjustment/ | S19 | (MH "Social Adjustment") |
| 19 | social behavior/ or aggression/ or social isolation/ or social marginalization/ or social skills/ or social stigma/ | 19 | social behavior/ or aggression/ or social isolation/ or social marginalization/ or social skills/ or social stigma/ | S20 | (MH "Social Behavior") OR (MH "Aggression") OR (MH "Social Isolation") OR (MH "Social skills") OR (MH "Stigma") |
|  |  |  |  | S21 | TI (“social marginali?ation”) OR AB (“social marginali?ation”) |
| 20 | employment/ or employment supported/ | 20 | employment/ or employment supported/ | S22 | (MH "Employment") OR (MH "Employment Supported") |
| 21 | personal autonomy/ | 21 | personal autonomy/ | S23 | (MH "Autonomy+") |
| 22 | Medication Adherence/ | 22 | Medication Adherence/ | S24 | (MH "Medication Compliance") |
| 23 | ((treatment or medication) adj compliance or adherence).tw. | 23 | ((treatment or medication) adj compliance or adherence).tw. | S25 | TI (treatment or medication W0 compliance or adherence) OR AB (treatment or medication W0 compliance or adherence) |
| 24 | (service adj (contact or u?e or utili?ation)).tw. | 24 | (service adj (contact or u?e or utili?ation)).tw. | S26 | TI (service W0 contact or use or utili?ation) OR AB (service W0 contact or use or utili?ation) |
| 25 | ((service or treatment) adj (satisfaction or quality)).tw. | 25 | ((service or treatment) adj (satisfaction or quality)).tw. | S27 | TI (service or treatment W0 satisfaction or quality) OR AB (service or treatment W0 satisfaction or quality) |
| 26 | (eviction or imprisonment or relapse or recall or move on or move-on or mental state).tw. | 26 | (eviction or imprisonment or relapse or recall or move on or move-on or mental state).tw. | S28 | TI (eviction or imprisonment or relapse or recall or "move on" or "move-on" or "mental state") OR AB (eviction or imprisonment or relapse or recall or "move on" or "move-on" or "mental state") |
| 27 | (social function* or recovery or empower* or esteem or self esteem or wellbeing or well being or well-being).tw. | 27 | (social function* or recovery or empower* or esteem or self esteem or wellbeing or well being or well-being).tw. | S29 | TI ("social function*" or recovery or empower* or esteem or "self esteem" or wellbeing or "well being" or "well-being") OR AB ("social function*" or recovery or empower* or esteem or "self esteem" or wellbeing or "well being" or "well-being") |
| *28* | (effectiveness or efficacy or outcome*).tw. | 28 | (effectiveness or efficacy or outcome*).tw. | S30 | TI (effectiveness or efficacy or outcome*) OR AB (effectiveness or efficacy or outcome*) |
| *29* | *12 or 13 or 14 or 15 or 16 or 17 or 18 or 19 or 20 or 21 or 22 or 23 or 24 or 25 or 26 or 27 or 28* | *29* | *12 or 13 or 14 or 15 or 16 or 17 or 18 or 19 or 20 or 21 or 22 or 23 or 24 or 25 or 26 or 27 or 28* | *S31* | *S13 OR S14 OR S15 OR S16 OR S17 OR S18 OR S19 OR S20 OR S21 OR S22 OR S23 OR S24 OR S25 OR S26 OR S27 OR S28 OR S29 OR S30* |
| **31** | **4 and 11 and 29** | **30** | **4 and 11 and 29** | **S31** | **S4 AND S12 AND S31** |
| **32** | **limit 30 to:**  **(humans and yr="1980 -Current" and ("young adult (19 to 24 years)" or "adult (19 to 44 years)" or "young adult and adult (19-24 and 19-44)" or "middle age (45 to 64 years)"))** | **31** | **Limit 30 to:**  **(human and yr="1980 -Current" and adult <18 to 64 years>)** | **S32** | **S31**  **(Limiters - Exclude MEDLINE records; Published Date: 1980.01.01-2015.12.31; Human; Age Groups: Adult: 19-44 years Middle Aged: 45-64 years)** |

| **IBSS (ProQuest)** | | **Sociological Abstracts (ProQuest)** | | **Applied Social Sciences Index and Abstracts (ProQuest)** | |
| --- | --- | --- | --- | --- | --- |
| *N* | *Terms* | *N* | *Terms* | *N* | *Terms* |
| **S1** | SU.EXACT.EXPLODE("Mental illness") | **S1** | SU.EXACT.EXPLODE("Mental illness") | **S1** | SU.EXACT.EXPLODE("Psychiatric disorders") |
| **S2** | SU.EXACT("Mental health") | **S2** | SU.EXACT.EXPLODE("Mental health") | **S2** | SU.EXACT.EXPLODE("Mental health") |
| **S3** | (psychol* OR psychiat* OR mental*) P/0 (illness* OR disorder* OR problem* OR disease* OR disab*) | **S3** | (psychol* OR psychiat* OR mental*) P/0 (illness* OR disorder* OR problem* OR disease* OR disab*) | **S3** | (psychol* OR psychiat* OR mental*) P/0 (illness* OR disorder* OR problem* OR disease* OR disab*) |
| **S4** | S1 OR S2 OR S3 | **S4** | S1 OR S2 OR S3 | **S4** | S1 OR S2 OR S3 |
|  |  |  |  | **S5** | SU.EXACT(“Group homes”) OR SU.EXACT (“Halfway houses”) OR SU.EXACT.EXPLODE(“Nursing homes”) |
| **S5** | ("residential facilit*") OR ("assisted living facilit*") OR ("group home*") OR ("halfway hous*") OR ("nursing home*") | **S5** | ("residential facilit*") OR ("assisted living facilit*") OR ("group home*") OR ("halfway hous*") OR ("nursing home*") | **S6** | ("residential facilit*") OR ("assisted living facilit*") |
| **S6** | “Residential treatment” | **S6** | “Residential treatment” | **S7** | SU.EXACT(“Residential treatment”) |
| **S7** | residential p/0 (care OR rehab* OR service* OR home) | **S7** | residential p/0 (care OR rehab* OR service* OR home) | **S8** | residential p/0 (care OR rehab* OR service* OR home) |
|  |  |  |  | **S9** | SU.EXACT(“Supported living”) OR SU.EXACT(“Sheltered housing”) OR SU.EXACT(“Sheltered accommodation”) |
| **S8** | (support* or shelter* or assist*) p/0 (hous* or home* or accom* or living or tenanc*) | **S8** | (support* or shelter* or assist*) p/0 (hous* or home* or accom* or living or tenanc*) | **S10** | (support* or shelter* or assist*) p/0 (hous* or home* or accom* or living or tenanc*) |
| **S9** | (floating or visiting) p/0 (support or outreach) | **S9** | (floating or visiting) p/0 (support or outreach) | **S11** | (floating or visiting) p/0 (support or outreach) |
| **S10** | outreach | **S10** | outreach | **S12** | outreach |
| ***S11*** | *S5 or S6 or S7 or S8 or S9 or S10* | ***S11*** | *S5 or S6 or S7 or S8 or S9 or S10* | ***S13*** | *S5 or S6 or S7 or S8 or S9 or S10 or S11 or S12* |
| **S12** | SU.EXACT("Hospitalization") | **S12** | SU.EXACT("Hospitalization") | **S14** | SU.EXACT("Hospitalization") |
| **S13** | SU.EXACT("Death") | **S13** | SU.EXACT("Death") | **S15** | SU.EXACT("Death") |
| **S14** | SU.EXACT("Suicide") | **S14** | SU.EXACT("Suicide") | **S16** | SU.EXACT("Suicide") |
| **S15** | SU.EXACT.EXPLODE("Satisfaction") | **S15** | SU.EXACT.EXPLODE("Satisfaction") | **S17** | SU.EXACT.EXPLODE("Satisfaction") |
| **S16** | "patient satisfaction" | **S16** | "patient satisfaction" | **S18** | "patient satisfaction" |
| **S17** | SU.EXACT("Quality of life") | **S17** | SU.EXACT("Quality of life") | **S19** | SU.EXACT("Quality of life") |
| **S18** | “social adjustment” | **S18** | “social adjustment” | **S20** | “social adjustment” |
| **S19** | SU.EXACT("Social behaviour") OR SU.EXACT("Aggression") OR SU.EXACT("Social isolation") | **S19** | SU.EXACT("Social behaviour") OR SU.EXACT("Aggression") OR SU.EXACT("Social isolation") | **S21** | SU.EXACT("Social behaviour") OR SU.EXACT("Aggression") |
| **S20** | “social marginali?ation” or “social skill*” or “stigma” | **S20** | “social marginali?ation” or “social skill*” or “stigma” | **S22** | “social isolation” or “social marginali?ation” or “social skill*” or “stigma” |
| **S21** | SU.EXACT("Employment") OR "supported employment" | **S21** | SU.EXACT("Employment") OR "supported employment" | **S23** | SU.EXACT(“Employment”) OR SU.EXACT(“Supported employment”) |
| **S22** | SU.EXACT("Autonomy") | **S22** | SU.EXACT("Autonomy") | **S24** | SU.EXACT("Autonomy") |
| **S23** | (medication or treatment) P/0 (adherence or compliance) | **S23** | (medication or treatment) P/0 (adherence or compliance) | **S25** | (medication or treatment) P/0 (adherence or compliance) |
| **S24** | service P/0 (contact or use or utili?ation) | **S24** | service P/0 (contact or use or utili?ation) | **S26** | service P/0 (contact or use or utili?ation) |
| **S25** | (service or treatment) P/0 (satisfaction or quality) | **S25** | (service or treatment) P/0 (satisfaction or quality) | **S27** | (service or treatment) P/0 (satisfaction or quality) |
| **S26** | eviction or imprisonment or relapse or recall or "move on" or "move-on" or "mental state" | **S26** | eviction or imprisonment or relapse or recall or "move on" or "move-on" or "mental state" | **S28** | eviction or imprisonment or relapse or recall or "move on" or "move-on" or "mental state" |
| **S27** | "social function*" or recovery or empower* or esteem or "self esteem" or wellbeing or "well being" or “well-being” | **S27** | "social function*" or recovery or empower* or esteem or "self esteem" or wellbeing or "well being" or “well-being” | **S29** | "social function*" or recovery or empower* or esteem or "self esteem" or wellbeing or "well being" or “well-being” |
| **S28** | effectiveness or efficacy or outcome* | **S28** | effectiveness or efficacy or outcome* | **S30** | effectiveness or efficacy or outcome* |
| ***S29*** | *S12 OR S13 OR S14 OR S15 OR S16 OR S17 OR S18 OR S19 OR S20 OR S21 OR S22 OR S23 OR S24 OR S25 OR S26 OR S27 OR S28* | ***S29*** | *S12 OR S13 OR S14 OR S15 OR S16 OR S17 OR S18 OR S19 OR S20 OR S21 OR S22 OR S23 OR S24 OR S25 OR S26 OR S27 OR S28* | *S31* | *S14 OR S15 OR S16 OR S17 OR S18 OR S19 OR S20 OR S21 OR S22 OR S23 OR S24 OR S25 OR S26 OR S27 OR S28 OR S29 OR S30* |
| **S30** | **S4 AND S11 AND S29** | **S30** | **S4 AND S11 AND S29** | **S30** | **S4 AND S13 AND S31** |

| **Cochrane Library** | | | | **Social Science Citation Index, SCI Expanded (Web of Science)** |
| --- | --- | --- | --- | --- |
| *N* | *N* | *Terms* | *N* | *Terms* |
| **1** | MeSH descriptor: [Mental Disorders] explode all trees | | **S1** | TS=("mental* disorder*" OR "mental* ill*" OR "mental* disease*" OR "mental problem*" OR "mental* disab*" OR "adjustment disorder*" OR "anxi* disorder" OR "stress disorder*" OR "mood disorder*" OR "affect* disorder" OR depress* OR bipolar OR "neurotic disorder*" OR "personality disorder*" OR schiz* OR psychos* OR psychot*) |
| **2** | MeSH descriptor: [Mental Health] this term only | | **S2** | TS= "mental* health*" |
| **3** | (psychol* or psychiat* or mental*) next (illness* or disorder* or problem* or disease* or disab*) | | **S3** | TS=((psycho* OR psychiatr*) NEAR/0 (ill* OR disorder* OR problem* OR disab* OR disease*)) |
| **4** | #1 or #2 or #3 | | **S4** | S1 OR S2 OR S3 |
| **5** | MeSH descriptor: [Residential Facilities] this term only | |  |  |
| **6** | MeSH descriptor: [Assisted Living Facilities] this term only | |  |  |
| **7** | MeSH descriptor: [Group Homes] this term only | |  |  |
| **8** | MeSH descriptor: [Halfway Houses] this term only | |  |  |
| **9** | MeSH descriptor: [Nursing Homes] this term only | |  |  |
|  |  | | **S5** | TS=(“residential facilit*” OR “assisted living facilit*” OR "group home*" OR "halfway house*" OR "nursing home*") |
| **10** | MeSH descriptor: [Residential Treatment] this term only | | **S6** | TS= “residential treatment” |
| **11** | residential next (care or rehab* or service* or home*) | | **S7** | TS=((residential) NEAR/0 (care OR rehab* OR service* OR home*)) |
| **12** | (support* or shelter* or assist*) next (hous* or home* or accom* or living or tenanc*) | | **S8** | TS=((support* OR shelter* OR assist*) NEAR/0 (hous* OR home* OR accom* OR living OR tenanc*)) |
| **13** | (floating or visiting) next (support or outreach) | | **S9** | TS=((floating OR visiting) NEAR/0 (support OR outreach)) |
| **14** | outreach | | **S10** | TS=outreach |
| **15** | #5 or #6 or #7 or #8 or #9 or #10 or #11 or #12 or #13 or #14 | | **S11** | S5 OR S6 OR S7 OR S8 OR S9 OR S10 |
| **16** | MeSH descriptor: [Hospitalization] explode all trees | | **S12** | TS=(hospitali?ation OR "hospital admission" OR readmission OR "hospital discharge" OR "length of stay") |
| **17** | MeSH descriptor: [Death] this term only | | **S13** | TS= death |
| **18** | MeSH descriptor: [Suicide] explode all trees | | **S14** | TS= suicide |
| **19** | MeSH descriptor: [Personal Satisfaction] this term only | | **S15** | TS= "personal satisfaction" |
| **20** | MeSH descriptor: [Patient Satisfaction] explode all trees | | **S16** | TS= ("patient satisfaction" OR “patient preference”) |
| **21** | MeSH descriptor: [Quality of Life] this term only | | **S17** | TS= “quality of life” |
| **22** | MeSH descriptor: [Social Adjustment] this term only | | **S18** | TS="social adjustment" |
| **23** | MeSH descriptor: [Social Behavior] this term only | | **S19** | TS= ("social behav*" OR aggression OR "social isolation" OR "social marginali?ation" OR "stigma" OR "social skill*") |
| **24** | MeSH descriptor: [Aggression] this term only | |  |  |
| **25** | MeSH descriptor: [Social Isolation] this term only | |  |  |
| **26** | MeSH descriptor: [Social Marginalization] this term only | |  |  |
| **27** | MeSH descriptor: [Social Stigma] this term only | |  |  |
| **28** | “Social skill*” | |  |  |
| **29** | MeSH descriptor: [Employment] this term only | | **S20** | TS= employment |
| **30** | MeSH descriptor: [Employment, Supported] this term only | |  |  |
| **31** | MeSH descriptor: [Personal Autonomy] this term only | | **S21** | TS= "personal autonomy" |
| **32** | MeSH descriptor: [Medication Adherence] this term only | | **S22** | TS= ((medication OR treatment) NEAR/0 (adherence OR compliance)) |
| **33** | (treatment or medication) next (compliance or adherence) | |  |  |
| **34** | service next (contact or use or utili?ation) | | **S23** | TS=((service) NEAR/0 (contact OR use OR utili?ation)) |
| **35** | (service or treatment) next (satisfaction or quality) | | **S24** | TS=((service OR treatment) NEAR/0 (satisfaction OR quality)) |
| **36** | eviction or imprisonment or relapse or recall or “move on” or “move-on” or “mental state” | | **S25** | TS=(eviction OR imprisonment OR relapse OR recall OR "move on" OR move-on OR "mental state") |
| **37** | “social function*” or recovery or empower* or esteem or “self esteem” or wellbeing or “well being” or “well-being” | | **S26** | TS=("social function*" OR recovery OR empower* OR esteem OR “self esteem” OR wellbeing OR "well being" OR “well-being”) |
| **38** | effectiveness or efficacy or outcome* | | **S27** | TS=(effectiveness OR efficacy OR outcome*) |
| ***39*** | *#16 or #17 or #18 or #19 or #20 or #21 or #22 or #23 or #24 or #25 or #26 or #27 or #28 or #29 or #30 or #31 or #32 or #33 or #34 or #35 or #36 or #37 or #38* | | ***S28*** | *S12 OR S13 OR S14 OR S15 OR S16 OR S17 OR S18 OR S19 OR S20 OR S21 OR S22 OR S23 OR S24 OR S25 OR S26 OR S27* |
| ***40*** | ***#4 and #15 and #39*** | | **S29** | **S4 OR S11 OR S28** |
| ***41*** | ***#4 and #15 and #39***  *Publication Year from 1980 to 2015* | | ***S30*** | **Refined by:** [excluding] **WEB OF SCIENCE CATEGORIES:** ( DENTISTRY ORAL SURGERY MEDICINE OR GERIATRICS GERONTOLOGY OR INFECTIOUS DISEASES OR VETERINARY SCIENCES OR GERONTOLOGY OR GENETICS HEREDITY OR COMPUTER SCIENCE INFORMATION SYSTEMS OR SURGERY OR RHEUMATOLOGY OR CLINICAL NEUROLOGY OR OBSTETRICS GYNECOLOGY OR GASTROENTEROLOGY HEPATOLOGY OR MEDICINE GENERAL INTERNAL OR TRANSPLANTATION OR ENDOCRINOLOGY METABOLISM OR TELECOMMUNICATIONS OR SUBSTANCE ABUSE OR PUBLIC ADMINISTRATION OR OPHTHALMOLOGY OR MEDICAL INFORMATICS OR MICROBIOLOGY OR PHARMACOLOGY PHARMACY OR CRITICAL CARE MEDICINE OR HORTICULTURE OR DERMATOLOGY OR COMPUTER SCIENCE ARTIFICIAL INTELLIGENCE OR PEDIATRICS OR ANESTHESIOLOGY OR CELL BIOLOGY OR EMERGENCY MEDICINE OR AUDIOLOGY SPEECH LANGUAGE PATHOLOGY OR EDUCATION SPECIAL OR VIROLOGY OR EDUCATION SCIENTIFIC DISCIPLINES OR PHYSIOLOGY OR OTORHINOLARYNGOLOGY OR NUTRITION DIETETICS OR PERIPHERAL VASCULAR DISEASE OR CRIMINOLOGY PENOLOGY OR ORTHOPEDICS OR ONCOLOGY OR RESPIRATORY SYSTEM OR IMMUNOLOGY OR BIOCHEMISTRY MOLECULAR BIOLOGY OR CARDIAC CARDIOVASCULAR SYSTEMS OR TROPICAL MEDICINE OR SPORT SCIENCES OR TOXICOLOGY OR INSTRUMENTS INSTRUMENTATION OR FOOD SCIENCE TECHNOLOGY OR UROLOGY NEPHROLOGY ) |

| **PsychInfo (OVID)** | |
| --- | --- |
| *N* | *Terms* |
| 1 | exp Mental Disorders/ |
| 2 | Mental Health/ |
| 3 | ((psychol* or psychiat* or mental*) adj (illness* or disorder* or problem* or disease* or disab*)).tw. |
| *4* | *1 or 2 or 3* |
| 5 | residential care institutions/ or assisted living/ or group homes/ or halfway houses/ or nursing homes/ |
| 6 | residential treatment.tw. |
| 7 | (residential adj (care or rehab* or service* or home*)).tw. |
| 8 | ((support* or shelter* or assist*) adj (hous* or home* or accom* or living or tenanc*)).tw. |
| 9 | ((floating or visiting) adj (support or outreach)).tw. |
| 10 | outreach.tw. |
| *11* | *5 or 6 or 7 or 8 or 9 or 10* |
| 12 | exp Hospitalization/ |
| 13 | “Death and dying”/ |
| 14 | exp Suicide/ |
| 15 | Satisfaction/ |
| 16 | exp Client Satisfaction/ |
| 17 | "Quality of Life"/ |
| 18 | Social Adjustment/ |
| 19 | social behavior/ or aggressive behavior/ or social isolation/ or marginalization/ or social skills/ or stigma/ |
| 20 | employment status/ or supported employment/ |
| 21 | personal autonomy.tw. |
| 22 | ((treatment or medication) adj compliance or adherence).tw. |
| 23 | (service adj (contact or u?e or utili?ation)).tw. |
| 24 | ((service or treatment) adj (satisfaction or quality)).tw. |
| 25 | (eviction or imprisonment or relapse or recall or move on or move-on or mental state).tw. |
| 26 | (social function* or recovery or empower* or esteem or self esteem or wellbeing or well being or well-being).tw. |
| 27 | (effectiveness or efficacy or outcome*).tw. |
| *28* | *12 or 13 or 14 or 15 or 16 or 17 or 18 or 19 or 20 or 21 or 22 or 23 or 24 or 25 or 26 or 27* |
| *29* | **4 and 11 and 28** |
| **30** | **limit 29 to:**  **(humans and yr="1980 -Current" and ("young adult (19 to 24 years)" or "adult (19 to 44 years)" or "young adult and adult (19-24 and 19-44)" or "middle age (45 to 64 years)"))** |
