# Supplementary material for: Mental health supported accommodation services: a systematic review of mental health and psychosocial outcomes
Source: BMC Psychiatry. 2018 May 15;18:128. doi: 10.1186/s12888-018-1725-8 (PMC5952646; doi:10.1186/s12888-018-1725-8)
Supplement: Supplementary file 2 — Summary table: All included studies. Summary of extracted data from all studies included in the final synthesis. (DOCX 60 kb) [file 12888_2018_1725_MOESM2_ESM.docx]

Additional File 2

Summary table: All included studies

| ***Author/year*** | ***Quality*** | ***Design/Follow-up*** | ***Subgroup*** | ***N=X*** | ***Housing type / Experimental groups*** | ***Outcomes assessed*** | ***Key findings*** |
| --- | --- | --- | --- | --- | --- | --- | --- |
| Anderson et al. (1993) | Moderate | Quasi-experimental  12 month follow-up | Deinstitutionalisation | N=556 | “Supported accommodation”   1. Long stay SUs who moved to different types of SA 2. Long-stay SUs who remained in hospital | 1. Psychiatric symptoms 2. Social functioning 3. Patient attitude ref. current placement and care | 1. No difference between groups 2. No changes in the size of social networks in both groups, the proportion of 'friends' as opposed to professionals in networks increased for Group 1. 3. More SUs in Group 1 than Group 2 expressed satisfaction with their situation (more leavers wanted to remain in current placement, fewer leavers said there was nothing they liked about their current place, more leavers found their medication helpful) |
| Baker F. & C. Douglas (1990) | Moderate | Cohort study  9 month follow-up | Deinstitutionalisation | N=844 | “Community support services”  None | 1. Unmet service needs 2. Global level of functioning 3. Maladaptive behaviour 4. QoL | 1. SUs in housing rated as appropriate had fewer unmet service needs than clients in living in inappropriate housing. Those in housing of below average physical conditions and housing inadequate for basic life activities had more unmet needs. 2. SUs in residences rated as adequate had improvement in functioning, SUs in “poor” adequacy had no change 3. SUs in housing with below average physical conditions showed a significant increase in the number of maladaptive behaviours 4. SUs moving from appropriate to inappropriate residential settings showed deterioration in their perceived QoL |
| Barbato et al. (2004) | Moderate | Cohort study  6 year follow-up | Deinstitutionalisation | N=337 | “Various community residences” | 1. Psychiatric symptoms 2. Social functioning 3. Social disability 4. Hospitalisation 5. Death 6. Accommodation stability | 1. No significant change over time 2. No significant change over time 3. The number of patients with mild or no disability increased significantly, whereas the number with moderate disability decreased significantly. 4. During the follow-up period, 35 of the discharged patients had at least one admission to an acute psychiatric ward, with a total of 79 admissions and 700 inpatient days, corresponding to a crude admission rate of 7%. 5. 22 patients died after discharge and before FU 6. At follow-up 112 patients (79% of the living patients) were still in the same place in which they had been accommodated at discharge, and 29 (21%) had moved at least once. |
| Benedikte et al (2002) | Moderate | Cohort study  10 year follow-up | Deinstitutionalisation | N=74 | “Halfway homes”  None | 1. Hospitalisation | 1. Duration of hospitalisation reduced over time. Residents were on average hospitalised 32% of the time before moving to the halfway houses. Through the 10 years after the stay residents were hospitalized, on average, 14% of the time. |
| Chan et al. (2003) | Moderate | Cohort study  1 year follow-up | Deinstitutionalisation | N=21 | “Halfway houses”  None | 1. QoL 2. Symptomology | 1. Significant improvement in QOL indices patients after discharged to HWHs. No significant changes in subjective QOL. The satisfaction with environment domain significantly decreased after the patient’s discharge to a HWH. 2. No significant improvement in BPRS total scores over the follow-up period |
| Chan et al. (2007) | High | Randomised control trial  2 year follow-up | Deinstitutionalisation | N=28 | “Supported group residence”   1. Supported Group Residence (SGR) 2. Group continuing in hospital | 1. Symptoms 2. Social function 3. Quality of life 4. General health quality 5. Medical/psychiatric cost | 1. Significant improvement in positive syndrome in the SGR group and a non-significant trend toward aggravation of negative syndrome in the hospital group. 2. The level of performance of the socially expected activities was more enhanced in the SGR group after 2 years. In addition, the level of both expectations for performance of social activities and performance of free-time activities assessed by the staff was also more enhanced in the SGR group. 3. Hospital group significantly declined in the physical domain. No differences were found in other domains. 4. There was no significant difference in general health between the two groups. No group differences between groups in medication dosage. 5. The expense of the hospital group was more than threefold greater than that of the SGR group. |
| Cohen & Hatib (1999) | Moderate | Case control  Unknown length of follow up | Deinstitutionalisation | N=105 | “Hostels” and “family homes”   1. Hostels 2. Family home | 1. Community tenure 2. Work 3. ADL | 1. Significantly better outcomes for Group 1 2. Significantly better outcomes for Group 1 3. Significantly better outcomes for Group 1 |
| Crosby et al. (2015) | Moderate | Cohort study  1 year follow up | Deinstitutionalisation | N=53 | Various residential settings  None | 1. Symptomology 2. Mental state 3. Quality of life | 1. Significant reduction in anergia at follow up. Significant increase in hostility-suspiciousness at follow-up. No significant change in BPRS. Significant reduction in negative symptoms: facial expression and spontaneous movements. Individual symptom ratings showed no significant changes, aside from reductions in blunted affect and emotional withdrawal. Significant reduction in depression levels. 2. Significant improvements in levels of social activity, speech skills and community skills. 3. Reported levels of life satisfaction in each of the life domains ranged from 66-93%, with highest levels of satisfaction being expressed in relation to living situation (93%). Global quality of life ratings showed no significant change over time. |
| Dayson et al. (1998) | Moderate | Cohort study  5 year follow-up | Deinstitutionalisation | N=17 | “Group homes”   1. Small, unstaffed house 2. Large, 24hr staffed house | 1. Social network | Group Home 1: At 1-year follow-up a cohesive group had emerged amongst the residents. This cohesive group was still apparent at 5 years follow up.  Group home 2: At 1-year follow-up the links between the residents were few and in general weak. At 5-year follow-up there were many more relationships between the men, but the vast majority of links were unreciprocated and the network lacked cohesion. |
| Dewees, Pulice & McCormick (1996) | Moderate | Cohort study  4 year follow-up | Deinstitutionalisation | N=46 | “Various community settings”  None | 1. Residential status 2. Symptomology 3. Hospitalisation 4. Access to medical care 5. Employment 6. Social support | 1. At follow up 34 clients, or 74 % had been placed in structured community housing. 33 participants (72 %) had maintained stability in community housing for the previous six months. 2. BPRS scores significantly higher among study participants who had spent a greater number of days in structured settings (100<, vs >100days) 3. 40 study participants (87 %) had been rehospitalized for some period of time. 23 participants (50 %) returned to hospital for cumulated periods of up to 1 year, and 17 (37%) returned for a year or more. SU who went to hospital were more likely to be residing in structured settings than in independent residential settings. 4. All sample had access, 28 clients (61%) relied on case managers to secure medical care. 5. None in the sample 6. Eighteen participants (39 %) had 3 sources of social support, while only one had none. 45 clients, or 98% had support from within the mental health system, and 35 clients, or 76 % had at least one support outside the system, including the family. Older SUs had significantly more support. |
| Duurkoop & VanDyck (2003) | Moderate | Cohort study  13 year follow-up | Deinstitutionalisation | N=174 | “Small scale facilities”   1. Congregate apartments (higher functioning SUs) 2. Residential care home (lower functioning SUs) | 1. Activities of daily living (ADL) 2. Psychiatric symptoms 3. Social network 4. Satisfaction with treatment and living conditions 5. Use of medication 6. Use of restrictive measures | 1. No change for Group1. Improvement for Group 2 2. No change for Group 1. Improvement for Group 2. 3. No change for either group. 4. Improvement for Group 1 and Group 2. 5. No change for either group. 6. Decrease in Group 2. |
| Eikelmann et al. (1993) | Moderate | Cohort study  5 year follow-up | Deinstitutionalisation | N=53 | “Half-way house”  None | 1. Residential status 2. Work status 3. Therapeutic needs 4. Social contacts 5. Hospitalizations | 1. Most patients moved into a supported group living home or (from a group living home) into their own flat. Only a small proportion returned to the psychiatric hospital. Three groups of SUs can be distinguished based on this outcome: 1.) those stable in their living situation status, i.e. continually moving toward more n=30. 2) those who stayed in the half-way house, 'fail' at the transition in a group living home or return following a temporary hospitalisation n=9 3) those who stay in the psychiatric hospital permanently n=6. 2. The number of types of paid employment had increased while the mean length of employment time per week reduced slightly 3. SUs used services ranging from in-patient stay to GP care at FU 4. There was a reduction in contact persons within the family and 'healthy' friends of more than half, while contacts with fellow patients increased. 5. Over the study period 2/3 of SUs were readmitted |
| Furlan et al. (2009) | High | Cohort study  4 year follow-up | Deinstitutionalisation | N=118 | “Community facilities”  None | 1. Symptomology 2. Autonomy 3. Relational skills | 1. Significant improvement at follow up. 2. Significant improvement at follow up. 3. Significant improvement on volition subscale. |
| Grinshpoon, Naisberg & Weizman (2006) | High | Cohort study  6 month follow-up | Deinstitutionalisation | N=92 | “Hostel-based residence”  None | 1. Psychiatric symptoms 2. Global functioning 3. Social functioning | 1. Significant reduction in most psychopathological symptoms, except for depression and severity of depression. 2. Significant decrease in Clinical Global Impression score and increase in GAF score. 3. Significant improvement in social competence and social interest as measured. Significant reduction in behaviour problems. Several significant positive changes in responses at follow-up on the Patients’ Attitude Questionnaire (PAQ). |
| Hadley et al. (1993) | Moderate | Cohort study  6 month follow-up | Deinstitutionalisation | N=382 | Various settings   1. Community residential rehabilitation (CRR) 2. Personal care boarding homes (PCBH) 3. Living with family/relative 4. Living alone/friend | 1. Community tenure | 1. SUs in PCBHs had longer community tenure than SUs living alone/with friend, no significant differences for the other pairwise comparisons.   Age and higher use of partial hospitalization best predicted the length of community tenure. |
| Higgins et al. (1997) | Moderate | Cohort study  11 month follow-up | Deinstitutionalisation | N=6 | “High-support community hostel” | 1. Levels of clinical functioning 2. Social functioning 3. Patients' attitudes towards their treatment settings 4. Need status and corresponding care interventions | 1. There were no significant differences between participants' first and second assessment scores for the mini-mental state examination, HADS, BPRS, SANS or the AIMS. 2. Social problems reduced over time. 3. Four residents still stated a preference for the hostel over hospital accommodation 4. There was a significant drop in the levels of unmet need for assessment of social skills. The total number of needs described as unmeetable needs also decreased significantly. |
| Kaiser et al. (2001) | Moderate | Quasi-experimental  5 year follow-up | Deinstitutionalisation | N=66 | “Supervised accommodation”  None | 1. Needs for care 2. Patients' assessment of treatment 3. Hospitalisation | 1. 61 % of the patients continued to live in highly staffed settings. Eleven percent had moved on to independent living and 16 % had returned into an institutional setting. 2. After one year patients (n=20) showed a significant increase in satisfaction with their living situation and more social contacts. 3. Average length of inpatient stay per year of the illness was decreased significantly at both follow-ups. |
| Kallert et al. (2007) | High | Cohort  2 year follow-up | Deinstitutionalisation | N=244 | “Various accommodation settings”  1. Nursing home in large psychiatric hospital  2. Social therapeutic hostel  3. Sheltered community residences  4. At home with family  5. Alone in own home | 1. Psychopathological symptoms (PANSS) 2. Social disabilities 3. Subjective QoL 4. Needs for care | 1. Significant deterioration in negative symptoms, general psychopathology and PANSS total score for Group 1. Significant improvement in positive symptoms for Group 2. No other significant changes in psychopathology. 2. Significant deterioration in global social adjustment for Group 1. 3. Significant reductions in general QoL for Group 1 and Group 2. 4. Group 1: Significant increase in clinical problems, clinical unmet needs, social problems and social unmet needs. Group 2: Significant increase in clinical problems. Group 5: Significant increase in clinical problems. |
| Lerner et al. (2012) | Moderate | Cohort study  N/A | Deinstitutionalisation | # of hospitalisations reported, not participants | “Various settings”  Study groups:  1. Hostels  2. Supportive  Housing  3. Vocational rehabilitation  Control groups:  4. Referred to  rehabilitation but did not implement the referral  5. Potential candidates for rehabilitation | 1. The probability of staying in the community after discharge from a hospitalization 2. Discharge | 1. The probability to stay out in the community at least one year was 58 to 74 percent for the different study groups without any rehabilitation history as compared to 22 to 39 percent in the control groups. After controlling for significantly contributing factors to rehospitalisation, a significantly higher probability was found for those placed in a rehabilitation program to stay in the community after discharge from the index hospitalization. 2. In the group without a prior history of rehabilitation, those patients who were placed in a (1) hostel or (2) supportive home after discharge from the index hospitalization had a probability of a significantly earlier discharge than both control groups. This was found to be true also for those with a past rehabilitation history. |
| Macmillan, Hornblow & Baird (1992) | Moderate | Cohort study  18 month follow-up | Deinstitutionalisation | N=69 | “Staffed houses”  None | 1. Social functioning 2. Symptomology 3. Use of resources 4. Relapse rate 5. Satisfaction with care 6. Impact on the community | 1. No significant change over time. 2. No significant change over time. 3. Significant reduction in visits per month to psychologist, social worker, and "other MH professionals" over time. No change in visits to other professionals. 4. Variable rates of relapse and rehospitalisation over time. No clear trends. 5. At each 6-month period, over 90% of residents stated that they were happy with their housing, had sufficient space to themselves, and sufficient help from staff. Contact with neighbours was generally poor, and over 50% reported no contact with friends during any of the three assessment periods. 6. Minimal complaints from community and very few law infringements. |
| Meehan et al. (2011) | Moderate | Cohort study  7 year follow-up | Deinstitutionalisation | N=181 | “Supported accommodation”  None | 1. Readmissions to long-term care 2. Readmissions to acute care 3. Death 4. Frequency of non-clinical support 5. Clinical functioning 6. QoL 7. Client satisfaction 8. Vocational outcomes 9. Forensic 10. Cost of care | 1. 4.9% readmitted to long term care 2. 60.2% readmitted to acute inpatient care at some point 3. 10.5% died 4. Significant reduction in the number of direct support hours per week 5. No significant change 6. No significant change 7. No significant change 8. 3.3% in paid employment 9. 2.7% committed offences 10. Supported housing cost less than acute inpatient care and community care unit. |
| Meissner (1998) | Moderate | Cohort study  3.7 year follow-up | Deinstitutionalisation | N=16 | “Halfway home”  None | 1. Hospitalization 2. Residential status 3. Work status | 1. SUs (50%) had hospital admissions during FU, average duration of stay 50.9 days – reduction in hospitalization. 2. Own flat was preferred over the accommodation managed by halfway house, proportion of SU in own flat increased with time. 3. Reduction of unemployment at the point of discharge, no further changes for the remaining FU period |
| Okin et al. (1995) | Moderate | Cohort study  4-10 year follow-up | Deinstitutionalisation | N=54 | “Structured community residential settings” | 1. Symptoms 2. Hospitalisations 3. Global functioning 4. Community tenure 5. Quality of life | 1. No significant change over time 2. 55% of patients required hospital readmission. The total sample spent 1.1 % of the time post-discharge in hospital. 3. Significant improvement in cognitive and social functioning 4. 57% of patients continued to live in structured community residential settings, 28% moved to independent living, and 16% returned to an institutional setting. 5. 94 % expressed a preference for life in the community. Patients’ mean ratings of their current quality of life on a 5-point scale were 3.75 for the total sample, 3.7 for patients with a diagnosis of schizophrenia, and 3.9 for patients with other diagnoses. |
| Salokangas et al (2006) | Moderate | Cohort study  3 year follow-up | Deinstitutionalisation | N=2221 | “Various accommodation settings”   1. Alone 2. With spouse or partner 3. With parents 4. With other person 5. In group home or dormitory 6. In institution 7. In temporary or other housing | 1. Life satisfaction | 1. Satisfaction at follow-up associated with female gender, good psychosocial functioning, having several confidants, or living in group homes or dormitories. Patients with depressive symptoms or with low psychosocial functioning and those who were currently hospitalized reported the smallest changes in levels of satisfaction between the two time points. |
| Shields, McGuinness & MacFlynn  (1995) | Moderate | Cohort study  6 month follow-up | Deinstitutionalisation | N=8 | “Residential care with nursing staff”  None | 1. Mental state 2. Social functioning / problems 3. Consumer satisfaction | 1. No significant difference in positive symptomatology. 2. No significant improvement in social functioning. Statistically significant improvement in the Problems Questionnaire. 3. On the measure of consumer satisfaction on Cantrill’ s Ladder, on a scale of 0 to 6, one subject rated satisfaction at 1, two subjects rated satisfaction exactly on the half-way mark 3, and the other five subjects rated greater than 3. |
| Trauer et al. (2001) | Moderate | Cohort study  1 year follow up | Deinstitutionalisation | N=125 | “Community care units”  None | 1. Symptoms 2. Personal functioning 3. Quality of life 4. Residential preferences 5. Aggressive behaviour 6. Social networks | 1. No significant change over time 2. No significant change over time 3. Significant increase in subjective satisfaction with living situation domain. No other significant change over time. 4. At follow-up, 4 SUs reported wanting to return to hospital. 5. 7 SUs had recorded aggressive behaviour during follow up period. 6. Small, significant increase in the mean number of persons having an emotional relationship with the patient and in satisfaction with co-residents |
| Trieman et al. (1998) | Moderate | Cohort study  5 year follow-up | Deinstitutionalisation | N=567 | Various accommodation settings  None | 1. Residential stability 2. Hospital admissions 3. Death | 1. 278 patients stayed in their original community placement. 27 patients moved from one house to another by way of intermediate hospitalization. Slight trend can be noted towards less supportive facilities. 2. 166 patients were readmitted to hospital at least once during follow-up period. Of these, 94 patients were admitted two or more times. 39 patients were in hospital at the end of the fifth year of follow-up. 3. 92 patients died during follow up period. Mortality and suicide rates were not significantly different to comparison group of patients in hospital |
| Vaslamatzis, Katsouyanni & Markidis  (1997) | Moderate | Cohort study  Up to 5 years | Deinstitutionalisation | N=59 | “Halfway house”  None | 1. Hospitalizations 2. Employment 3. Living independently | 1. Not rehospitalized after discharge (19 persons); one or two hospitalizations not exceeding 60 days (11 persons); remained in hospital for a period of more than 60 days (11 persons). 2. Seven subjects were working during the whole follow-up period; 16 had not worked at all; 18 were working at some time during the follow-up period. 3. 15 of the 41 (36%) were living independently during follow-up. |
| Casper & Clark (2004) | High | Case-control study  1 year follow-up | General SMI | N=56 | “Supportive housing program”  1. Forensic  2. Non-forensic | 1. Service utilization 2. Incidents 3. Hospitalizations | 1. The forensic consumers had utilized significantly more services during the first year of their residence and for each of the four quarters of that year. 2. A history of incarceration was significantly associated with 911 calls as well as all other incidents combined. 3. Receiving specialized CM services was the only significant predictor for hospitalizations. |
| Boydell & Everett  (1992) | Moderate | Cohort study  1 year follow-up | General SMI | N=14 | “Supported housing”  None | 1. Social climate 2. Formal vs informal support 3. Intensity of contact | 1. After 1 year, staff and tenants perceived environment to be above average in areas of involvement, support, spontaneity, autonomy, practical orientation and personal problem orientation and below average in staff control. At TP1 staff felt that there was more emphasis on autonomy, personal problem orientation and open expression of anger tenants felt there was more emphasis on staff control. At TP2 the differences lessened (only significant difference on emphasis on expression of anger). 2. Over time staff perceived reduction of SU involvement, tenants reported increase in the extent to which they were encouraged to understand their feeling and personal problems. No significant difference in the number of social supports between TP1 and TP2. 3. There was a decrease of 59.7% in staff time spent on service delivery over 1 year (greatest decrease in the areas of support, recreation and leisure and documentation) |
| Clarkson et al. (1999) | Moderate | Cohort study  6 month follow-up | General SMI | N=39 | “Community support worker”  None | 1. Unmet needs 2. QoL 3. Satisfaction 4. Social functioning 5. Symptomology 6. Service usage 7. Cost | 1. Keyworker ratings suggest an overall fall in the number of unmet needs and in the proportion of needs which are unmet and a rise in the number of met needs and in the number of overall needs. According to patient ratings, the number of both met and unmet needs had risen over the study period, with little change in the proportion of needs met. 2. No significant change over time. 3. No significant change over time. 4. Social networks decreased significantly. 5. Significant decreases in social problems in two domains, and a trend towards a lower overall number of problems. 6. No significant change over time. 7. The receipt of the CSW service did not lead to a significant extra total cost of services overall. |
| D’Avanzo et al. (2004) | High | Cohort study  1 year follow-up | General SMI | N=1792 | “Community residential facilities”    None | 1. Move-on | In the study period, a total of 316 patients were discharged. Among these, 191 (11%) went to lower-protection settings or home and 49 (3%) to higher-protection settings. The probability of discharge to lower-protection settings and home was higher for people in residential care, not coming from a psychiatric hospital, having shorter duration of the current admission, having work at the time of admission and with a low HoNOS score.  Associations were found between discharge to higher-protection settings and old age, inadequate accommodation in staff opinion, and the public sector managing the facility. |
| de Girolamo et al. (2014) | Moderate | Cohort study  1 year follow-up | General SMI | N=403 | “Residential care”  None | 1. Psychopathology 2. Social functioning 3. Psychosocial functioning and disability 4. Cognitive function 5. Physical health 6. Residential status | 1. Statistically significant improvement for SUs discharged to own home. 2. No significant changes over time 3. Statistically significant improvement on work skills sub-scale for SUs discharged to own home 4. No significant changes over time 5. No significant changes over time 6. 104 (25.8 %) patients were discharged: 55 discharged to home, 33 to other RFs, 9 to supported housing, and 6 to prison. For 16, unknown outcome. 10 patients died before 1-year follow up. |
| de Mooij et al. (2016) | Moderate | Cohort study  6 year follow-up | General SMI | N=323 | “Residential settings”   1. Sheltered housing 2. Independent housing | 1. Hospital admissions 2. Residential mobility | 1. The severity of negative symptoms predicted rehospitalisation and duration of hospitalisation. Disorganisation symptoms predicted the duration of hospitalisation. 2. Half the patients stayed in the same care setting. Twenty-one per cent of SMI patients changed care setting once, 11% twice, 5% three times, and 14% four or more times. Residential changes in were more frequent than changes in care setting: 78% changed address one or more times. Twenty-three per cent of patients with SMI changed address once, 19% twice, 10% three times and 26% four times or more. |
| Grant & Westhues (2010) | High | Cohort study  1 year follow-up | General SMI | N=27 | “Mental health supported housing”  1. High support  2. Low support | 1. Satisfaction with housing 2. Satisfaction with social support 3. Mental health 4. Physical health 5. Mastery 6. Taking medications as prescribed 7. Hospitalizations | After Bonferroni adjustments:   1. No significant change for either group. 2. No significant change for either group. 3. No significant change for either group. 4. Significant change in perceptions of physical health for high support group. No significant change for low support group. 5. No significant change for either group. 6. No significant change for either group. 7. No significant change for either group. |
| Guazzelli et al. (2000) | High | Cohort study  2 year follow-up | General SMI | N=19 | “Family-style, residential, community-based program”  None | 1. Level of staff assistance (complete / partial / minimal) 2. Symptomology 3. Negative symptoms 4. Functional impairment | 1. 7/19 moved from complete to minimal assistance; 6/19 moved from complete to partial assistance; and 6/19 remained at the complete level. One of the seven residents who moved to the minimal-assistance level also moved outside the rehabilitation community. 2. As a group, the residents showed reduction in overall psychiatric symptoms of more than 40 percent. 3. Over the two-year period, patients’ negative symptoms showed a 20 percent improvement as measured by the total score on the SANS 4. 40 percent reduction in impairment. |
| Hanrahan et al. (2001) | Moderate | Cohort study  2 year follow-up | General SMI | N=74 | “Community  integrated living arrangements”  1.Intermittent-care residents  2.Continuous-care residents | 1. Satisfaction with services  2. Hospitalisation | 1. No significant differences in satisfaction scores were found between groups. No demographic differences in satisfaction scores were found. 2. The mean number of days in hospital decreased significantly from baseline to the first year in community living arrangement facilities. No difference between groups. |
| Hawthorne, Fals-Stewart & Lohr  (1994) | Moderate | Cohort study  1 year follow-up | General SMI | N=104 | “Community-based residential care” | 1. Number of hospital admissions 2. Number of days in hospital 3. Employment status 4. Living status 5. Global Assessment of Functioning (GAF) Scale | 1. Significant reduction in mean number of hospital and crisis centre admissions. 2. Significant reduction in number of hospital and crisis centre days 3. Compared with their status at program entry, a significantly greater proportion of the residents were employed at one year follow-up. 4. Compared with their status at program entry, a significantly greater proportion of the residents were living independently and significantly fewer were homeless, at one year follow-up. 5. Significant increase in mean GAF scones |
| Hodgins, Cyr & Gaston  (1990) | Moderate | Case control  2 year follow-up | General SMI | N=112 | “Supervised apartments”   1. Supervised living apartments 2. Independent living | 1. Symptomatology 2. Psychosocial functioning 3. Stress 4. Social support 5. Quality of life 6. Life satisfaction 7. Hospital admissions 8. Outpatient appointments | 1. Group 1 showed more thought disorder at follow up. No significant difference in depression scores. 2. No significant group differences 3. No significant group differences 4. No significant group differences 5. No significant group differences 6. No significant group differences 7. No significant group differences 8. No significant group differences |
| Law et al.  (2002) | Moderate | Cohort study  1 year follow-up | General SMI | N=179 | “Long stay care homes”  None | 1. Quality of life 2. Social disability | 1. Significant increase in QoL from baseline to follow up. 2. Significant improvement in social disability from baseline to follow up. |
| Lora et al. (2004) | Moderate | Cohort study  12 month follow-up | General SMI | N=83 | “Community residential facilities”   1. Discharged patients 2. Long-term patients | 1. Global functioning 2. Symptomology 3. QOL 4. Family burden | 1. Significant Improvement over time 2. Significant reduction in positive symptoms over time. No change in negative symptoms. Group 2: 3. Group 1: Significant improvement over time. Group 2: No significant change over time. 4. No change |
| Malinovsky et al. (2013) | Moderate | Quasi-experimental  Unsure of exact follow-up period. | General SMI | N=627 | “Various accommodation settings”  None | 1. Hospitalisation rates 2. Self-report indicators of recovery 3. Indicators of staff competency 4. Processes that promote recovery | 1. The total number of days spent in the hospital by residents decreased by 40%. 2. Staff reports: Significant improvement in domains of health, adaptation, behaviour and global functioning   Resident reports: No statistically significant improvements on any domain of the MCAS or the global rating.   1. Staff self-ratings: Statistically significant improvements on three of the seven subscales - client preferences, holistic approach, and optimism. 2. Senior staff reports: Statistically significant improvements on all aspects of the working alliance. There was also a significant improvement in the overall rating of working alliance by senior staff. Resident’s reports: No significant change in working alliance. No significant change in the level of hope among residents. |
| McDermott et al. (2016) | Moderate | Cohort study  2 year follow-up | General SMI | N=197 | “Permanent supported housing” | 1. Hospitalisation 2. Duration of hospitalisations | 1. Hospital use decreased for SUs in the programme (sustained over time) 2. SUs had shorter mental health hospital admissions after joining the program |
| Middleboe (1997) | Moderate | Cohort study  Av. 1.1 year follow-up | General SMI | N=47 | “Small group homes”  None | 1. Psychopathology 2. QoL 3. Social Integration 4. Social Network 5. Social functioning 6. Hospitalisation Index | 1. Significant reductions in neurotic, depressive and psychotic symptoms, and overall psychopathology. 2. Significant increase in QoL 3. Significant increase in social integration. 4. No significant change in overall social network, but significant increase in number of reciprocal supportive contacts. 5. No significant change in overall social functioning 6. Significant reduction in hospitalisation rates |
| Muir et al. (2008) | Moderate | Cohort study  18 month follow-up | General SMI | Varied (N=54-69) | “Supported housing”  None | 1. Housing Tenancy 2. Mental Health 3. Personal Wellbeing 4. General Health 5. Access to Services 6. Community Participation | 1. 70% of SUs maintained stable housing. 15% moved properties (either to exit the program or to move into more appropriate housing) 2. 84% of SUs spent less time in hospital, compared to the immediate years prior to joining the program. 12% percent spent a greater proportion of time in hospital since participating in the program. Overall, 81% reduction in days hospitalised. 3. A slight decrease in overall satisfaction in wellbeing between the TP1 and TP3. 4. 60% of SUs reported improved physical health 5. 96% of SUs accessed clinical mental health professionals, psychiatrists and general practitioners 6. Statistically significant increases in participation included paid and voluntary work, training and education, as well as social and community activities. |
| Muir et al. (2010) | Moderate | Cohort  2 year follow-up | General SMI | N=55 | “Supported housing” | 1. Social network 2. Work/education participation | 1. Significant increase in established friendships 2. Significant increase in work (paid, voluntary, supported or open) and/or education/training participation. |
| Nelson et al (1998) | Moderate | Cohort study  12 month follow-up | General SMI | N=173 | Various accommodation settings  None | 1. Emotional well being 2. Independent functioning | 1. The size of a person’s peer network was directly related to positive affect. Housing concerns and not having one’s own room were directly related to negative affect. 2. The number of residents in participants’ housing was inversely related to their levels of independent functioning. Resident control was directly related to residents’ level of independent functioning |
| Sakiyama et al. (2002) | Moderate | Cohort study  6.5 year follow-up | General SMI | N=55 | “Support house”   1. Recovery group (patients able to live alone or with family) 2. Re-hospitalization group (patients who were later re-hospitalized due to difficulty adapting to the support house). | 1. Residential status | 1. Group 1 had a significantly shorter total hospitalization period and a smaller number of hospitalizations before entry into the facility, compared to Group 2. Group 1 was significantly better than Group 2 in measures of thought disorder. No significant difference between the groups on total BPRS score, at the time of entry into the facility. Group 1 significantly better than Group 2 in measures of ‘speech skills’ and ‘self-care’ at the time of entry into the facility. Group 2 experienced a relapse of, mainly positive, symptoms, at the time of the discontinuation of the use of the facility or re-admission. |
| Segal & Holschuh  (1991) | Moderate | Cohort study  10-12 year follow up | General SMI | N=360 | “Supported housing”   1. Supportive sheltered care 2. Transitional, high-expectation sheltered care | 1. Social network size 2. Social network / types of relationships | 1. Significantly smaller network size in Group 2. Larger network size for SUs who had been in sheltered care during FU. 2. Group 2 less likely to receive emotional support, give emotional support and receive instrumental support. |
| Segal & Kotler (1993) | Moderate | Cohort study  10 year follow-up | General SMI | N=393 | “Sheltered-care facilities”  None | 1. Social functioning 2. Physical health status 3. Psychopathology 4. Employment 5. Death | 1. No significant change in self-initiated social interactions at follow up. No significant change in assisted social interactions. When aging accounted for, results show a reduction in independent social functioning and an increase in assisted social functioning 2. Poorer health reported at follow up. When aging accounted for, symptoms decreased. 3. No significant difference at follow up. When aging accounted for, symptoms decreased. 4. Workforce participation was significantly related to prior paid work experience 5. Significantly higher mortality rate compared to general population (2.85 times the rate) |
| Shu et al.  (2001) | Moderate | Cohort study  6 month follow-up | General SMI | N=60 | “Half-way house”  1. Half-way house  2. Home care | 1. QoL 2. Autonomy 3. Social activity 4. Life satisfaction 5. Health maintenance 6. Family support 7. Economic function 8. Physical health 9. Psychological wellbeing | 1. The overall QOL in patients receiving home care service was significantly higher than those receiving half way house service when adjusted for age, sex, education, number of admission, duration of symptom and the time interval. 2. The autonomy of patients receiving home care was significantly better than in the half-way house care group. However, autonomy did not change over time. 3. Patients receiving home care had significantly higher scores in social activity compared to those patients receiving half-way house services. 4. No significant difference between the groups 5. No significant difference between the groups 6. No significant difference between the groups 7. No significant difference between the groups 8. No significant difference between the groups 9. No significant difference between the groups |
| Caplan et al. (2006) | Moderate | Randomised control trial  2 year follow-up | Homeless | N=118 | Group housing vs. independent apartments   1. Evolving consumer households (ECH) 2. Independent apartments (IA)   Subgroups: Substance abusers vs non-abusers | 1. Executive functioning 2. Verbal memory 3. Attention | 1. Non-abusers: Perseverations improve between TP1 and TP2 in the ECH housing, and then at a lesser rate until Time 3. In the IA housing, by contrast, total perseverations worsen over time. Abusers: Except for an initial reduction in the ECH housing until TP2, perseverations remain stable among the substance abusers. 2. Scores tend to improve over time, with little systematic variation in the magnitude of change between housing types or substance abuse status. 3. Scores tend to improve over time, with little systematic variation in the magnitude of change between housing types or substance abuse status. |
| Dickey et al. (1996) | Moderate | Quasi-experimental  18 month follow-up | Homeless | N=112 | “Supported housing”  1. Single-occupancy apartments  2. Evolving consumer households | 1. Service use 2. Residential status | 1. No significant group differences. More than 50 % of the clients in Group 2 were hospitalized, and 40% used emergency crisis services. 2. SUs in Group 2 were housed 92 % of the time; SUs in Group 1 were housed 83 % of the time. Number of days hospitalized was negatively associated with housing stability. Strong association between higher housing stability and less service use. |
| Gabrielian et al. (2016) | Moderate | Case control study  12 month follow-up | Homeless | N=102 | “Transitional housing” | 1. Completion vs premature exit | 1. SUs who exited prematurely were more likely to be on parole at enrolment, to have active substance use disorders, diagnosis of chronic pain, or HCV, alcohol and drug disorders, to have one or more inpatient medical-surgical admissions (26% versus 8%) and used more emergency department services (75% versus 45% with one or more visits). Stayers had significantly better adherence to outpatient medical and mental health care. |
| Gilmer et al. (2010) | Moderate | Quasi-experimental  2 year follow-up | Homeless | N=363 | “Full-service partnerships” (FSPs)   1. FSP clients 2. Control Group | 1. Residential status  2. Access to financial support  3. Employment  4. Mental health service use  5. Quality of life | 1. Significant decline in days homeless in Group 1 2. Significant increase in the percent of clients receiving disability benefits 3. No significant changes 4. Significantly larger increase in case management visits per year, medication management visits per year, therapy/rehabilitation per year, and total visits per year in Group 1. Significant decrease in use of inpatient, emergency, and justice system services in Group 1 5. Better QoL in Group 1 |
| Gilmer et al. (2014) | Moderate | Cohort study  2 year follow-up | Homeless | N= 6584 | “Permanent supported housing” (Housing First)   1. High HF fidelity projects 2. Medium HF fidelity projects 3. Low fidelity projects | 1. Residential status | 1. After adjustment for individual characteristics, days spent homeless after enrolment declined by 87 for Group 1 and 34 for Group 3. After adjustment for days spent homeless before enrolment, days spent homeless after enrolment declined by 63 for Group 1 and by 53 for Group 3. After enrolment, clients in for Group 1 spent more than 60 additional days in apartments than clients in Group 3. |
| Gilmer et al. (2014a) | Moderate | Quasi-experimental  2 year follow-up | Homeless | N= 10231 | “Full-service partnerships” (FSPs)   1. FSP group 2. Matched control group | 1. Service use. 2. Costs | 1. The mean annual number of mental health outpatient visits increased by 55.5 in Group 1. 2. Annual mental health costs increased by US$11,725 relative to the matched control group. Total service costs increased by US$12,056. |
| Henwood et al. (2014) | Moderate | Cohort study  1 year follow-up | Homeless | N=80 | “Permanent Supportive Housing”  None | 1. QoL | 1. Subjective satisfaction with living situation, family relations and finances were all significantly increased at 12 months. Time in independent housing remained significantly associated with three of the quality of life domains. Community participation was significantly related to frequency of social contacts. Symptom severity was significantly related to 4 QoL domains and negatively related to QoL in all six models; indicating that psychiatric symptom severity negatively and broadly impacts quality of life. |
| Henwood et al. (2015) | Moderate | Cohort study  12 month follow-up | Homeless | N=75 | “Permanent supported housing”   1. Treatment first (TF) 2. Housing first (HF) | 1. Met needs 2. Type of needs | 1. Significantly more HF than TF SUs had their baseline needs met. 2. The average number of deficiency needs identified by HF and TF SUs did not differ at baseline, but at 12-months significantly more TF SUs identified the need for housing and employment as compared to HF SUs. |
| Hurlburt et al. (1996) | Moderate | Randomised control trial  24 month follow-up | Homeless | N=362 | “Permanent supported housing”   1. Comprehensive case management (CCM) with access to a S8 certificate 2. Traditional CM with access to a S8 certificate 3. CCM without access to a S8 certificate 4. Traditional CM without access to a S8 certificate | 1. Housing consistency 2. Housing stability | 1. Housing type and case management style had no effect on housing consistency 2. Clients with CCM were no more likely to achieve stable housing or stable independent housing than clients with only traditional CM. SUs in Groups 1 and 2 (Section 8 certificate) were more likely to achieve stable independent living arrangements, stabilize in independent housing faster and less likely to drop out of the social service program than those without access to a certificate. |
| Kasprow et al. (2000) | High | Cohort  One year follow up | Homeless | N=65424 (Veterans contacted by VA homeless program)  N=35792 (Eligible for HUD-VA SH program) | “Supported housing”  None | 1. Successful move through the HUD-VA supported housing program steps 2. Housing retention at 1 year follow up or at the time of discharge from case management (if before 1 year) | 1. Of the 2,058 veterans who obtained a Section 8 voucher, 1,800 (87.5 percent) subsequently selected and moved into an apartment. The median time between obtaining the voucher and moving into an apartment was 37 days. No veterans’ characteristics were significantly related to apartment attainment. Veterans were more likely to attain an apartment if the HUD-VA program case manager accompanied them to the public housing authority on more than one occasion. 2. Of the 1,800 veterans who obtained an apartment, 1,649 were eligible for analysis of one-year housing status; one year had not yet elapsed for the remaining 151 veterans. Of the veterans eligible for this analysis, 83.9 percent were housed. Only one veteran characteristic, gender, predicted one-year housing status. Women were significantly more likely than men to be housed. Case managers’ efforts to secure Supplemental Security Income on the veteran’s behalf were positively related to being housed at one year (OR=1.53, CI= 1.14 to 1.92). |
| Lee et al.  (2009) | Moderate | Cohort study  30 month follow-up | Homeless | N=237 | “Supported independent living program”  None | 1. Residential status | 1. 69% of SU maintained continuous residence in the program for the study period, 14% experienced a positive departure, and 17% a negative departure. Past substance abuse increased the probability of a negative departure, and a more supportive relationship with program staff decreased the probability. A higher income increased the probability of a positive departure, whereas a higher level of social distress in the neighbourhood decreased the probability. |
| Lipton et al. (2000) | High | Cohort study | Homeless | N=2937 | “Supportive housing”  1. High intensity settings  2. Moderate intensity settings  3. Low intensity settings | 1. Tenure in housing | 1. Low-intensity settings: 54% continuously housed after five years. Moderate-intensity settings: 56% continuously housed after five years. High-intensity settings: 37% continuously housed after five years. Individuals placed in high-intensity settings accounted for most of the increased hazard of becoming discontinuously housed during the first 120 days after placement. |
| Mares & McGuire  (2000) | Moderate | Cohort study  4 year follow-up | Homeless | N=321 | “Board-and-care homes”   1. Community residential care program (received monthly home visits from case managers) 2. Comparison group (no visits) | 1. Hospitalisations | 1. Group 1 demonstrated a significant reduction in median number of psychiatric bed-days. No significant change for comparison subjects. Group 2 subjects were hospitalized 1.7 times more often than Group 1 subjects. Younger subjects were hospitalized 1.8 times more often than older subjects. |
| McCarthy et al. (2013) | Moderate | Cohort study  4 year follow-up | Homeless | N=281066 (including *n*=106 suicide deaths) | “Nursing homes” | 1. Death from suicide within 6 months of live discharge | 1. Suicide rates within 6 months of discharge were 88.0 per 100000 persons. Standardized mortality ratios relative to age- and gender-matched individuals in the VA patient population were 2.3 for men and 2.4 overall. Resident characteristics, diagnoses, behaviours, and pain were not significantly associated with suicide risk. |
| McHugo et al. (2004) | High | Randomised control trial  18 month follow-up | Homeless | N=121 | “Supported housing”  1. Parallel housing services (CM and housing  services provided by single agency)  2. Integrated housing services (CM services provided by mobile AO teams and housing by community-based landlords) | 1. Residential status  2. Symptomology  3. Quality of life  4. Substance use  5. Victimisation | 1. Both programs reduced functional homelessness and increased the amount of time in stable housing but Group 2 showed significantly greater gains. No significant group differences in portion of days in one's own apartment or proportion of time in institutional settings. 2. Significantly less severe psychiatric symptoms in Group2 3. Group gender interaction, with males in Group 1 reporting significantly lower QoL. 4. No significant group differences 5. No significant group differences |
| Metraux et al. (2003) | Moderate | Cohort study  24 month follow-up | Homeless | N=6334 | “Homeless shelter”   1. Shelter users with housing placement 2. Shelter users without housing placements | 1. Use of homeless shelter | 1. SUs who received the housing placement used, on average, 128.2 fewer shelter days than those in the control group (statistically significant difference). |
| Montgomery et al. (2017) | Moderate | Cohort study  Unspecified follow-up period | Homeless | N=20146 | “Permanent supportive housing” (HUD-VASH programme) | 1. Completion vs premature exit | 1. Evicted SUs were more often male, not receiving disability related compensation, have chronic medical condition, higher prevalence of suicidal and self-harm behaviours, and shorter time in the HUD-VASH programme, higher utilization rates for outpatient services. Significantly larger proportion of evicted SUs had at least one inpatient admission or emergency service visit. Evicted SUs had contact with case managers during the 31–90 days prior to exit, but not 0–30 days prior to exit. |
| Newman et al. (1994) | High | Cohort study  36 month follow-up | Homeless | N=299 | “Independent living”  None | 1. Hospitalization 2. Residential stability 3. Service needs | 1. Greater neighbourhood quality was associated with longer hospital stays. No effect for change in number of hospitalizations per month. 2. In one of the two research sites positive and significant change in housing problems had an effect on residential stability (less moves). 3. Housing problems and neighbourhood problems (positive and significant) had an effect on service needs. Positive correlation between housing problems and service needs, negative between neighbourhood problems and service needs.   For SU who stayed longer than 18m: Affordability is associated with reduction in hospital days/month. |
| Patterson et al. (2013) | Moderate | Randomised controlled trial  1 year follow-up | Homeless | N=497 | “Supported housing”   1. High needs (HN)   1a. Congregate housing (HFCONG)  1b. Indep. housing with outreach (HFACT)  1c. Treatment as usual (TAU)   1. Moderate needs (MN)   2a. Indep. housing with CM (HFICM)  2b. Treatment as usual (MNTAU) | 1. QoL | 1. After 6 and 12 months of participation in the study, HF participants reported significantly greater overall QoL as compared to TAU regardless of the type of housing and support they received and regardless of their level of need. HF participants reported stronger feelings of safety in their neighbourhoods, and comfort and satisfaction with their living arrangements at both 6 and 12 months post-baseline. There was no significant effect of housing type on overall or subscale scores. |
| Patterson et al. (2014) | Moderate | Randomised control trial  12 month follow-up | Homeless | N=497 | Various settings   1. Housing First with Assertive Community Treatment (ACT) 2. Congregate Housing with on-site support (CONG) 3. High Needs Treatment as Usual (HN-TAU) 4. Housing First with Intensive Case Management (ICM) 5. Moderate Needs Treatment as Usual (MN-TAU) | 1. Psychological community integration 2. Physical community integration | 1. Improvement at 6 and 12 months for the ICM group compared to MN-TAU. No significant improvement over time for either of the ACT or CONG groups compared to HNTAU 2. No change over time, no difference between groups |
| Schutt et al. (2007) | Moderate | Quasi-experimental  18 month follow- up | Homeless | N=112 | “Supported housing”  1. Single-occupancy apartments  2. Evolving consumer households | 1. Community Functioning | 1. Better baseline executive functioning predicted improved self-care and behavioural turbulence ratings for respondents in Group 1 compared with those in Group 2. The number of positive social contacts reported rose for subjects with better baseline verbal memory living in Group 2 but not in those in Group1. |
| Seidman et al. (2003) | High | Randomised control trial  18 month follow up | Homeless | N=112 | “Housing for homeless mentally ill persons”   1. A group home 2. Independent apartments | 1. Neuropsychological impairment score 2. Executive functioning | 1. Neuropsychological functioning improved significantly among the entire study sample. Significant improvements in delayed verbal memory, motor speed and sequencing. 2. Executive functioning significantly declined among the participants who were assigned to independent apartments. The effect of housing type was statistically significant. |
| Siegal et al. (2006) | High | Quasi-experimental  18-month follow-up | Homeless | N=139 | “Supported housing” and “Community residences”   1. Supported housing 2. Community residences | 1. Residential stability 2. Housing satisfaction 3. Emergency department and crisis service use 4. Personal well-being 5. Overall Choice and Empowerment Scale 6. Quality of Life 7. Symptomology | 1. No significant group differences 2. Greater satisfaction in Group 1 3. Significantly less use of crisis services in Group 1 (“true stayers” analysis) 4. No significant difference at follow up 5. Lower for Group 1 only in 3 propensity strata (SU most likely to be placed in supported housing) 6. No significant differences 7. No significant differences |
| Stergiopoulos et al. (2014) | High | Cohort study  6 month follow-up | Homeless | N=301 | “Housing First”  None | 1. Community Integration –Physical 2. Community Integration - Psychological 3. Symptomatology 4. Substance Use 5. Community Functioning 6. Quality of Life | 1. 60% participants followed the expected trajectory of improvement. Participant-rated working alliance score was positively associated with changes from baseline. 2. 62% participants followed the expected trajectory of improvement. Participant-rated working alliance score was positively associated with changes from baseline. 3. 71% participants followed the expected trajectory of improvement. Diagnosis of alcohol or substance abuse or dependence was associated with a reduction in mental illness symptomatology. 4. 72% participants followed the expected trajectory of improvement. Diagnosis of alcohol or substance abuse or dependence was associated with a negative change in problems due to substance use. 5. 67% participants followed the expected trajectory of improvement 6. 66% participants followed the expected trajectory of improvement. Diagnosis of psychotic disorder was associated with negative changes from baseline to 6-months in the quality of life domain scores. Participant-rated working alliance score and diagnosis of alcohol or substance abuse or dependence was associated with a positive change from baseline in quality of life. |
| Stergiopoulos et al. (2015) | Moderate | Randomised control trial  24 month follow-up | Homeless | N=378 | “Permanent supported housing”   1. Permanent supported housing (Housing First) with ICM 2. TAU | 1. Housing stability 2. Physical and mental health 3. Substance use problems 4. Social functioning 5. Quality of life 6. Health care utilisation | 1. Group 1 spent a significantly higher percentage of time in stable residences compared to those in Group 2 2. The intervention did not result in any significant treatment x time interactions in either physical health or mental illness symptomatology at either 12 or 24 months 3. In Group 1 compared to Group 2, at 12m, no significant difference at 24m. Significant reduction of number of days spent experiencing alcohol problems among Group 1 compared to Group 2 at 12 and 24m. Significant reduction of money spent on alcohol at 24m. 4. Community functioning improved significantly in Group 1 compared to Group 2 at 24-months. No difference between groups in terms of psychological and physical community integration 5. Improvement for Group 1 as compared to Group 2 at 6-months, but no difference at 24-months. 6. No significant differences in mean number of hospital days or number of emergency service visits.   Ethnicity had a significant main effect on physical health, severity of substance use related problems, number of emergency department visits, amount of money spent on alcohol and drugs and the number of days experiencing problems due to alcohol and drug use. In all associations, greater improvement was observed among racialized participants, compared to non-racialized participants. |
| Tsai & Rosenheck (2012) | Moderate | Cohort study  1 year follow-up | Homeless | N=734 | “Supported housing for homeless”  None | 1. Number of days housed and type of accommodation 2. Psychiatric symptoms 3. Quality of life 4. Community integration | 1. Domains of consumer choice assessed at three months were not predictive of housing outcomes at six or 12 months. 2. Choice over living environment at three months associated with the BSI measure of subjective distress. 3. Choice over living environment at three months associated with subjective quality of life at six and 12 months. 4. Choice over CM at three months associated with community integration at 12 months. |
| Tsai, Mares & Rosenheck (2012) | Moderate | Cohort study  2 year follow-up | Homeless | N=384 | “Multisite  supported housing” | 1. Housing satisfaction 2. Functional outcomes 3. Subjective outcomes | 1. Six domains of housing satisfaction were identified: good environment, control and consumer choice, physical quality, geographic proximity to desirable resources, positive case manager contact, and frequency of landlord interaction. Over the follow-up period, there were slight declines in housing satisfaction on physical quality of housing and satisfaction with case manager contact, and an increase in satisfaction with landlord interaction. 2. Housing satisfaction was not predictive of functional outcomes (community integration, addiction severity, employment, nights spent in own accommodation) 3. Housing satisfaction was not found to be predictive of subjective outcomes (QoL, social support, choice in treatment, symptoms, therapeutic alliance) |
| Tsai, Mares & Rosenheck (2012a) | Moderate | Cohort  1 year follow-up | Homeless | N=550 | “Supported housing”  None | 1. Mental/physical health 2. Symptomology 3. Substance misuse 4. Housing 5. Social support 6. Work and income 7. Community participation 8. Civic activity 9. Religious faith | 1. Small but statistically significant improvement was observed on the SF-12 mental health subscale. No change in physical health. 2. Small but significant reduction in symptomology. 3. Significant reduction in ASI alcohol subscale. 4. After housing placement significantly more nights in their own apartment and significantly fewer nights in institutions or homeless. 5. No significant change over time. 6. The number of participants who did volunteer work and the average number of hours volunteered significantly declined over time. 7. Participants also showed a small but statistically significant increase in their community participation over time. 8. Participants also showed a small but significant increase in civic activity. 9. Participants also showed a significant decrease in how important they rated their religious faith.   NOTE: When analyses of social integration were repeated controlling for measures of baseline clinical status and change in clinical symptoms over time, all significant changes in social integration were no longer significant except for those in the housing domain. |
| Tsemberis & Eisenberg (2000) | Moderate | Cohort  Up to 5 years follow-up | Homeless | N=1841 | “Housing First”   1. Pathways (Housing First) 2. Residential treatment programme (continuum of care) | 1. Housing retention | 1. 88 % of those in group 1 and 47 % of those in the comparison group remained housed. Type of program was the second most important predictor of housing tenure. Being older or having a mood disorder increased tenure in housing, whereas having a dual diagnosis and being white decreased housing tenure. |
| Tsemberis et al. (2003) | High | RCT  6 month follow-up | Homeless | N=225 | “Supported housing”   1. Pathways 2. Linear continuum of care | 1. Housing stability 2. Days homeless 3. Symptomology 4. Self-esteem 5. QoL 6. Substance use 7. Alcohol Use | 1. Group 1 spent more time in stable housing. Group 2 spent more time in institutional and transitional placements. 79% of Group 1 was living in stable housing 6 months after baseline compared to 27% of Group 2. 2. Group 1 were less likely to be homeless. Days homeless before baseline was associated with days homeless afterwards. The experimental intervention was most effective in reducing homelessness for participants with the most extensive prior histories of homelessness. 3. No significant difference between groups at 6 months. 4. No significant difference between groups at 6 months. 5. No significant difference between groups at 6 months. 6. No significant difference between groups at 6 months. 7. No significant difference between groups at 6 months. |
| Wood et al. (1998) | High | Quasi-experimental  3 year follow-up | Homeless | N=316 | “Supported housing”  1. Section-8 certificate and comprehensive CM  2. Section-8 certificate and traditional CM  3. Comprehensive CM without  access to Section-8  4. Traditional CM without access to Section-8 | 1. Housing stability 2. Mental health 3. Substance misuse 4. Family support | 1. After two years, 61% of participants had stable housing. Those in the program with access to Section 8 certificates were more likely to have stable housing at 2 years (67.3%) than those without access to Section 8 certificates (53.7%) 2. Significant improvement in mental health. 3. No significant change in substance misuse 4. Significant improvement in satisfaction with family, perceived availability of family and frequency of interactions. Improvement in family support was not associated with housing or case management conditions. |

** CM = Case management; QoL = Quality of life; HWH = Half-way house; TP = Time point; HUD-VASH = Housing and Urban Development ― Veterans Affairs Supportive Housing Program; SU = Service user; RF = Residential facility; ADL = Activities of daily living; AO = Assertive outreach; TAU = Treatment as usual
